# Analogies and Differences in the Photoactivation Mechanism of Bathy and Canonical Bacteriophytochromes Revealed by Multiscale Modeling

**DOI:** 10.1021/acs.jpclett.4c01823

**Published:** 2024-08-01

**Authors:** Giacomo Salvadori, Benedetta Mennucci

**Affiliations:** † Institute for Computational Biomedicine (INM-9/IAS-5), Forschungszentrum Jülich, 52428 Jülich, Germany; ‡ Dipartimento di Chimica e Chimica Industriale, University of Pisa, 56124 Pisa, Italy

## Abstract

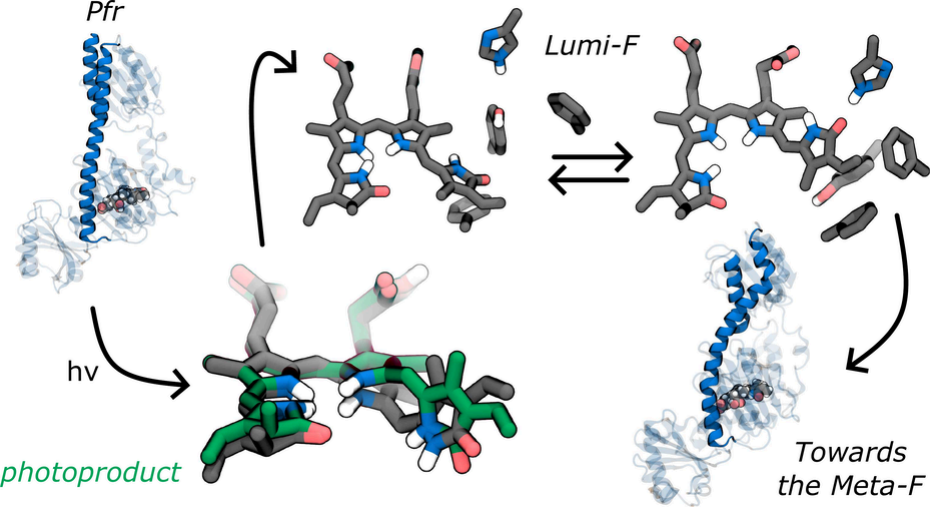

Bacteriophytochromes are light-sensing biological machines that switch between two photoreversible states, Pr and Pfr. Their relative stability is opposite in canonical and bathy bacteriophytochromes, but in both cases the switch between them is triggered by the photoisomerization of an embedded bilin chromophore. We applied an integrated multiscale strategy of excited-state QM/MM nonadiabatic dynamics and (QM/)MM molecular dynamics simulations with enhanced sampling techniques to the *Agrobacterium fabrum* bathy phytochrome and compared the results with those obtained for the canonical phytochrome *Deinococcus radiodurans*. Contrary to what recently suggested, we found that photoactivation in both phytochromes is triggered by the same hula-twist motion of the bilin chromophore. However, only in the bathy phytochrome, the bilin reaches the final rotated structure already in the first intermediate. This allows a reorientation of the binding pocket in a microsecond time scale, which can propagate through the entire protein causing the spine to tilt.

Phytochromes are photoreceptors found in plants, fungi, and bacteria that regulate fundamental biological processes using light.^1−8^ They are homodimeric soluble proteins which consist of a multidomain apoprotein and a photoswitchable bilin chromophore (Figure 1), which is covalently anchored to the protein via a thioether linkage with a cysteine residue. A hallmark feature of phytochromes is that they can adopt two photoreversible forms with distinct spectroscopic properties: the Pr state, which absorbs red light, and the Pfr state, which absorbs far-red light.^9−12^ These states differ in protein structure and bilin stereochemistry, which is ZZZssa for Pr and ZZEssa for Pfr.

**Figure 1:**
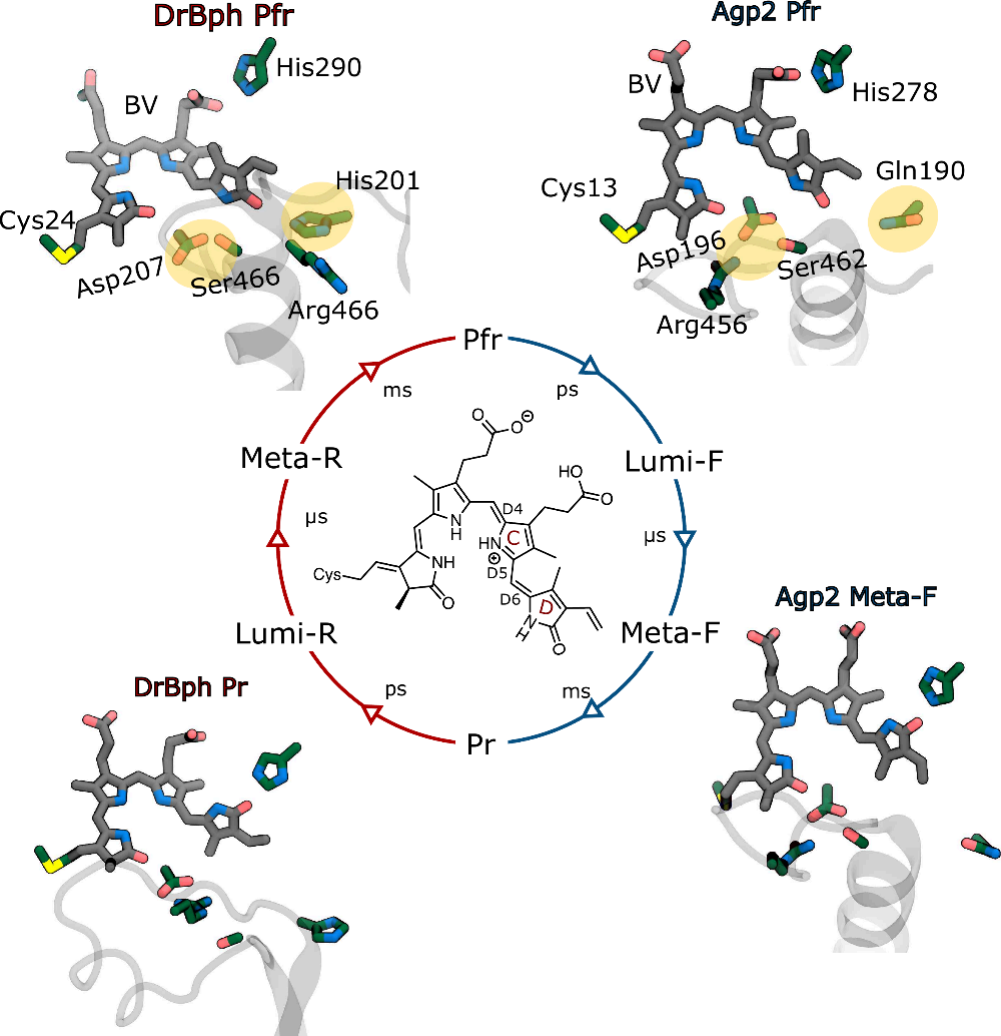
Photocycle and the experimental structural evidence. In canonical and bathy phytochromes, Pr and Pfr are the stable dark states, respectively. After light absorption, canonical phytochromes follow the red pathway, while bathy phytochromes follow the blue pathway. A close-up view of BV and the nearby residues is shown for the crystallographic structures of Pr (PDB ID: 4Q0J(22)) and Pfr (PDB ID: 5C5K(23)) forms in DrBph, and Pfr (PDB ID: 6G1Y(24)) and Meta-F (PDB ID: 6G20(24)) forms in Agp2. The chemical structure and definition of the main dihedral angles for the embedded biliverdin are also shown.

Phytochromes are classified into two groups: canonical and bathy. Canonical phytochromes, such as the *Deinococcus radiodurans* bacteriophytochrome (DrBph), have the Pr form as their resting state. In contrast, bathy phytochromes, such as the *Agrobacterium fabrum* bacteriophytochrome (Agp2), have the Pfr form as their thermodynamically stable state.^13−15^ The increased stability of the Pfr state in bathy phytochromes is not due to a different chromophore (biliverdin IXα, BV, in both Agp2 and DrBph), but instead to the different composition and shape of the binding pocket.^16,17^ The latter also determines the protonation state of the C-ring propionic group (Cprop, Figure 1), which infrared difference spectroscopy showed to be protonated in bathy phytochromes due to an unprecedentedly high p*K*_a_.^16,18−20^ The larger stability of the Pfr state in Agp2 has been connected to the presence of a salt-bridge interaction between Asp196 and Arg456 (Figure 1), which allows a stable hydrogen bond between the aspartate residue and the NH group of the D ring in the ZZEssa stereochemistry of BV. Moreover, the D ring also interacts with a glutamine residue in Agp2, whereas a histidine residue is present at the same site in DrBph (Gln190 and His201 in Figure 1).

In both canonical and bathy phytochromes, the photoactivation is initiated by a light-induced isomerization around the double bond of the bilin chromophore, which connects the D and C rings (D6 in Figure 1). This local change is then propagated to the binding pocket and subsequently to the entire apoprotein, which undergoes large conformational changes comprising a secondary structure transition of the tongue between an α-helical and a β-sheet structure (Figure S7).^21^

Thanks to vibrational spectroscopic techniques,^25^ we know that along the inactive-to-active transition, phytochromes pass through at least two spectrally distinguishable intermediate states (Figure 1),^26−30^ namely Lumi(-R/F) and Meta(-R/F), although the exact number varies from phytochrome to phytochrome. This is because the D-ring-carbonyl stretching mode is very localized and environmentally sensitive, therefore it can be used as an excellent probe to follow changes in the interactions of the chromophore with the nearby residues. For DrBph, there are several clues to the mechanism leading to the early Lumi-R structure coming from both femtosecond X-ray crystallographic data^31^ and computational simulations.^32,33^ These data strongly support a counterclockwise rotation of the D-ring on the picosecond time scale, following a kinetics affected by a hydrogen bond between the D-ring carbonyl group and a conserved histidine (His290 in DrBph). The resulting early Lumi-R intermediate is characterized by the BV having a structure intermediate, for what concerns the rotation of the D ring, between those found in the Pr and Pfr states, respectively. This early intermediate evolves on the microsecond time scale into a late intermediate characterized by a much more disordered binding pocket but with the same structure of the bilin.^30,32^ For Agp2, a recent spectroscopic study^34^ has instead proposed a different mechanism. According to this study, the photoisomerization is preceded by a transient deprotonation of the D (or C) ring into a hydrogen-bonded water cluster.

In order to confirm or exclude these differences in the photoactivation mechanism of the two types of bacteriophytochromes, we have applied to Agp2 the same multiscale strategy previously used for DrBph.^32^ Such a strategy, outlined in the Supporting Information (Figure S1), integrates ground-state (QM/) MM molecular dynamics (MD) simulations and excited-state QM/MM nonadiabatic dynamics with enhanced sampling techniques to cover the multiple time scales from the ultrafast picosecond scale of the photochemical event, up to the significantly slower microsecond scales of the protein conformational changes.^35^ A detailed comparison between the two phytochromes reveals that, contrary to previous literature, the photoisomerization mechanism remains consistent. However, significant differences in the binding pocket of the two phytochromes cause variations in the propagation of structural changes from the pigment to the intermediates along the multistep process leading to their respective active states.

We started the simulations by exploring the configurational space of the Pfr state of Agp2 by running four MM-MD replicas of 2 μs each of the system in aqueous solution. In all cases, the initial structural model was generated starting from the 6G1Y^24^ entry of the Protein Data Bank. All the details of the MD simulations are reported in the Supporting Information. Along the four trajectories, the resting Pfr state of Agp2 adopts a nearly parallel spine geometry (Figure S7a), whereas in the canonical DrBph phytochrome, the active Pfr state has a Y-framed geometry (Figure S7b).^21,36^

Analyzing the binding pocket, we can see how the Asp196 residue is strongly stabilized by hydrogen bonds with Arg456, Ser462, and Tyr251, respectively (Figure S8). We recall that in the Pfr of DrBph, only the Ser462 interacts with the same aspartate, and the corresponding arginine residue is far from the binding pocket (Figure 1).^23^ As a result, the aspartate residue can establish a more stable interaction with the D ring of BV, which is further stabilized by a hydrogen bond (direct or water-mediated) with Gln190 (Figure S8). To capture the heterogeneity around BV we have used a principal component analysis (PCA) based on intermolecular distances involving the D ring and the nearby residues in the chromophore binding pocket (see Supporting Information for more details). On top of that, we have applied a hierarchical clustering algorithm by identifying three clusters, labeled 0, 1, and 2, that differ mainly in the interactions between D ring and the protein (Figure S2).

As reported before, a recent spectroscopic study^34^ has suggested that the D-ring rotation is preceded by an ultrafast proton transfer from the excited bilin to a network of water molecules. A proposed mechanism posits that the proton is transferred from the NH to the carbonyl group in the D ring, either directly or via a transient protonation of the nearby Asp196, and then to a network of water molecules. The chromophore is protonated back only once it reaches the ground state of the Lumi-F intermediate.

To investigate the proton-transfer hypothesis, we performed ground-state and excited-state QM/MM optimizations of BV before (keto-like) and after (enol-like) the proton transfer from N–H to C = O of D ring (Figure 2). From this analysis, we found an energy difference between the keto-like and enol-like forms of the order of 27 kcal mol^–1^. This result is confirmed also if we consider the case of a first proton transfer from the D ring to Asp196 (Figure 2, Tab. S1 and section S2).

**Figure 2:**
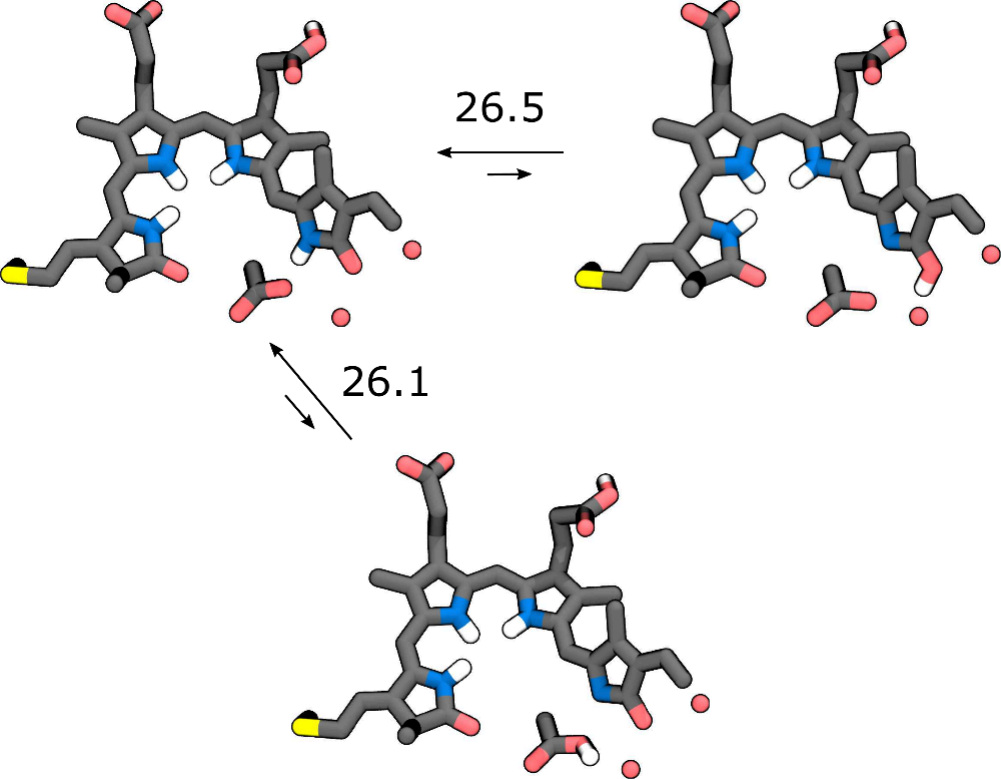
Excited state proton transfer. Representative structures of the keto and enolic forms, and after the proton transfer to the aspartate residue, together with the corresponding energy difference with respect to the keto form. All values in kcal mol^–1^.

We further investigated the proton-transfer mechanism by switching to a QM/MM dynamic description. To accelerate the sampling, we relied on the On-the-fly Probability Enhanced Sampling (OPES) method,^37,38^ using as a collective variable the difference in the proton distance from the D-ring nitrogen and oxygen (section S2). The results obtained for ten different trajectories fully confirm what found with the static picture, namely an extremely unfavorable proton transfer process.

Once having ruled out the proton-coupled photoisomerization, we focused on the nonadiabatic dynamics following the excitation of BV.

We randomly extracted ten configurations from the three clusters obtained in the sampling of the Pfr state (Figure S2) and run ten ground-state QM/MM MD trajectories. The configurations and momenta extracted from these trajectories were then used as initial conditions for the nonadiabatic dynamics simulation through the surface hopping (SH) method.^39−41^ A total of 3028 SH trajectories were run. These were all initialized with BV in its lowest (S_1_) excited state. For these simulations, we considered only the first three singlet states, since due to the ultrafast decay to the ground state found in pump–probe experiments,^34^ triplet states should not be involved. More details on the SH trajactories are reported in the Supporting Information.

Our nonadiabatic simulations show that photoisomerization can indeed occur without a proton-coupled mechanism. In fact, the photoproduct is obtained with a quantum yield of 28% following the same mechanism found for DrBph.^32,33^ All trajectories, regardless of the initial value of the D5 and D6 dihedral angles, follow the same mechanism, which is characterized by a counterclockwise (ccw) isomerization of the D6 dihedral angle associated with a concomitant clockwise (cw) isomerization of the adjacent D5 dihedral angle (Figure 3a). This hula-twist motion has been found to be the isomerization coordinate in other photoactive proteins,^42−45^ as it allows a complete rotation of the double bond, minimizing steric interactions with protein pocket residues. In fact, by analyzing the initial and final structures of the SH trajectories, we observe a small variation in the D-ring orientation (Figure 3b).

**Figure 3:**
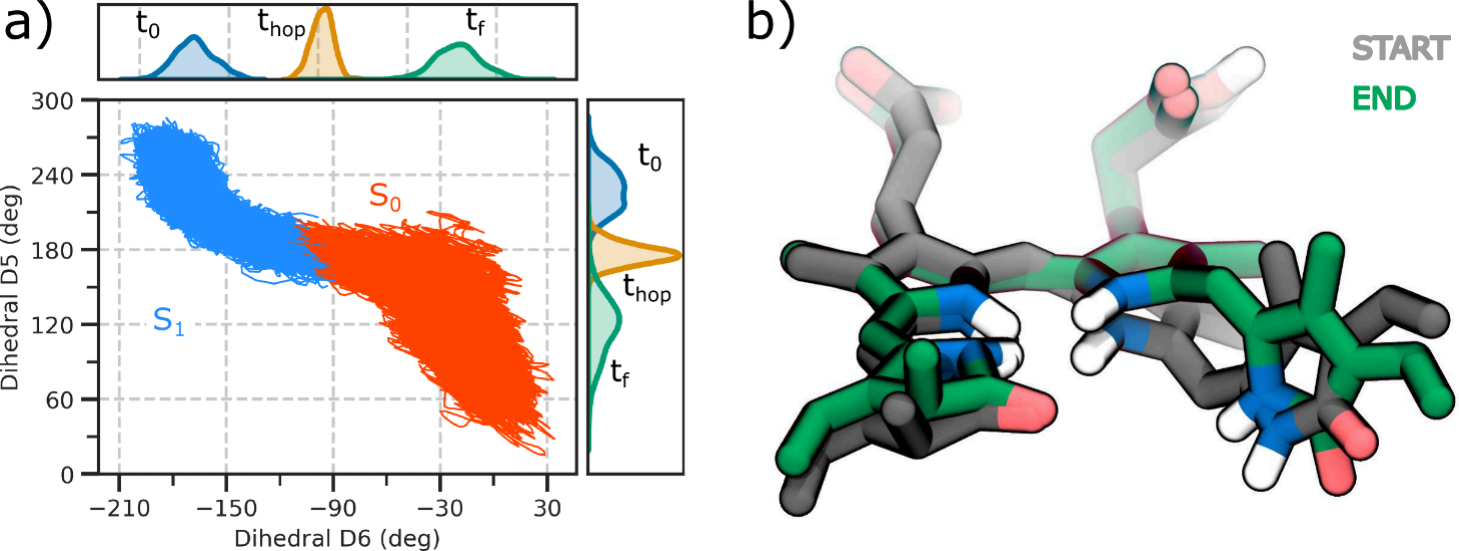
Excited state dynamics. a) Correlation between D5 and D6 dihedral angles. All reactive trajectories are represented by blue lines for those running on S_1_, and red lines for S_0_. The density distribution is shown at the starting conditions, at the S_1_ → S_0_ hop, and at the end of the simulation. b) Superimposition of two representative structures of BV at the beginning (gray) and end (green) of one SH simulation to highlight the hula-twist mechanism.

The large heterogeneity in the BV-protein interactions found in the ground state is reflected in the variability of decay times (Figure S9): there are short-lived trajectories that decay in 100 fs, and long-lived ones that are still in the excited state after 20 ps (61 trajectories out of 3028). Overall, the S_1_ state exhibits a biexponential decay, with a fast component that decays in 0.71 ps and a slow component that decays in 4.25 ps (Figure S11). This biexponential decay is in agreement with what found experimentally for the bathy bacteriophytochrome from *Pseudomonas aeruginosa* (PaBph) through fs-resolved fluorescence and absorption methods.^46^ By analyzing how the lifetimes change in the heterogeneous configurations of the system, we found that long lifetimes are observed when there is a strong interaction between NH in D ring and Asp196, and at least one water molecule is coordinated to the CO in the same ring (Figure S10). In contrast, all structures where the interaction of D-ring carbonyl with water molecules is replaced by that of Gln190 exhibit short excited-state lifetimes (Figure S10).

To characterize the evolution of the photoproduct, we run 63 1.5 ns-long ground-state QM/MM MD simulations starting from the final structures of the SH reactive trajectories (more details are reported in the Supporting Information).

In all trajectories, the D ring continues the ccw rotation. However, the D5 dihedral angle only reaches its final (Pr) value in 20 out of 63 simulations, forming a hydrogen bond between the D-ring carbonyl and either a histidine residue, His278, or a tyrosine residue, Tyr165 (Figure 4a,b and Figure S12). This scenario will now be referred to as BV–Pr. We note that, in contrast to DrBph, where the same ccw rotation led to a steric clash between two methyl groups,^32^ here the clash is between a methyl group and a hydrogen, which is energetically easier to overcome. In the other trajectories, instead, BV does not reach a Pr-like structure, but it remains in an intermediate structure between Pr and Pfr with respect to the D5 and D6 dihedral angles (BV–Pr′ from here on). This is a result which resembles what observed for DrBph.^32^ In only two trajectories a planar structure is reached (BV–Pr″, from here on) (Figure S12). In all cases, the relaxation of the photoproduct does not significantly affect the other dihedral angles, except for D4 and D6, which change by about 15 degrees (Figure S13).

**Figure 4:**
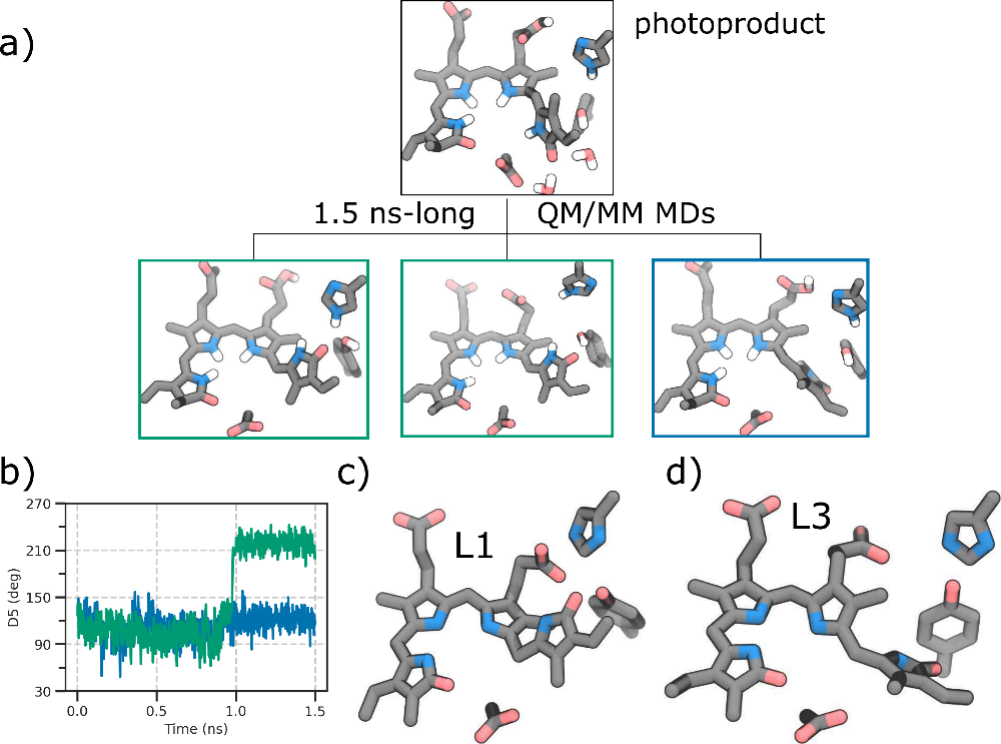
Evolution of the photoproduct. a) Representative structures of the BV and the nearby residues in the photoproduct, and after 1.5 ns-long QM/MM MD simulations. The two green boxes refer to the BV–Pr scenario involving the hydrogen bond with the His278 or the Tyr165 residue. The blue box refers to the BV–Pr′ scenario. b) Trend of the D5 dihedral angle along different trajectories associated with the structures in the panel a). c), d) Experimental intermediates, L1 (PDB: 3NOP) and L3 (PDB: 3NOU), respectively, obtained through temperature-scan cryo-crystallography for the bathy phytochrome PaBph.^47^

The observed BV–Pr and BV–Pr′ structures are very close to the L1 and L3 forms of the Lumi-F intermediate found in a temperature-scan cryo-crystallography experiment for the bathy phytochrome PaBph (Figure 4c,d).^47^

To extend the time evolution on the μs-time scale, we extracted ten different structures (comprising of BV–Pr, BV–Pr′, and BV–Pr″) from the QM/MM MDs and used them as starting points for μs-long MM MDs.

In the first nanoseconds, trajectories from both BV–Pr′ and BV–Pr″ completed the rotation of the D ring, reaching the BV–Pr structure (Figure S14) which is stabilized by a hydrogen bond interaction between the D-ring carbonyl and Tyr165 (Figure S15d-f, Figure S15). However, over the time scale of hundreds of nanoseconds, a dynamic behavior between BV–Pr and BV–Pr′ is found in three out of ten trajectories (Figure S14). This dynamic behavior is controlled by the heterogeneity of the environment, especially around the protonated Cprop (Figure S16). In fact, this group exhibits a significantly enhanced mobility compared to the DrBph phytochrome in which the same group is deprotonated^32^ (Figure S17). To investigate the role of the protonation state of Cprop, we run a 1 μs-long MM MD trajectory starting again from BV–Pr′ but deprotonating Cprop and protonating the His278 residue. The trajectory shows that the deprotonated BV switches almost immediately to a BV–Pr structure (D5 in Figure S18) and it remains in such a geometry thanks to a stabilizing hydrogen bonding interaction with the His278 residue (Figure S19). Moreover, the mobility of the deprotonated Cprop is now much more limited due to its strong interactions with His248, Ser260, and Ser262 (Figure S19). These findings suggest that a deprotonation of the Cprop group to the nearby histidine eliminates the heterogeneity that characterizes the chromophore in the Lumi-F intermediate.

In two trajectories from BV–Pr, we observe structural rearrangements in the neighboring residues of the chromophore binding pocket, particularly Tyr165, Phe187, and Phe192, which adopt an arrangement similar to the one found in the Meta-F intermediate (Figure 5a).^24^ Now, the His278 residue interacts strongly with the D-ring carbonyl (Figure 5a,d, Figure S15). These rearrangements are correlated with the positional shift of the Gln190 residue, which in the initial Pfr structure was closer to the BV chromophore (Figure 1). This correlation is further confirmed by another trajectory starting from the BV–Pr where the Gln190 remains close to the D ring and we do not observe any structural rearrangement, being the bilin strongly stabilized by the Tyr165 residue (Figure 5b,e). We can connect these different behaviors to the position of the Trp440 residue which is far away from the Gln190 residue in the BV–Pr showing rearrangements (Figure 5a), but very close to it in the BV–Pr not showing rearrangements (Figure 5b). In the latter case, it hinders Gln190 displacement and the consequent rearrangements of the binding pocket toward the Meta-F intermediate.

**Figure 5:**
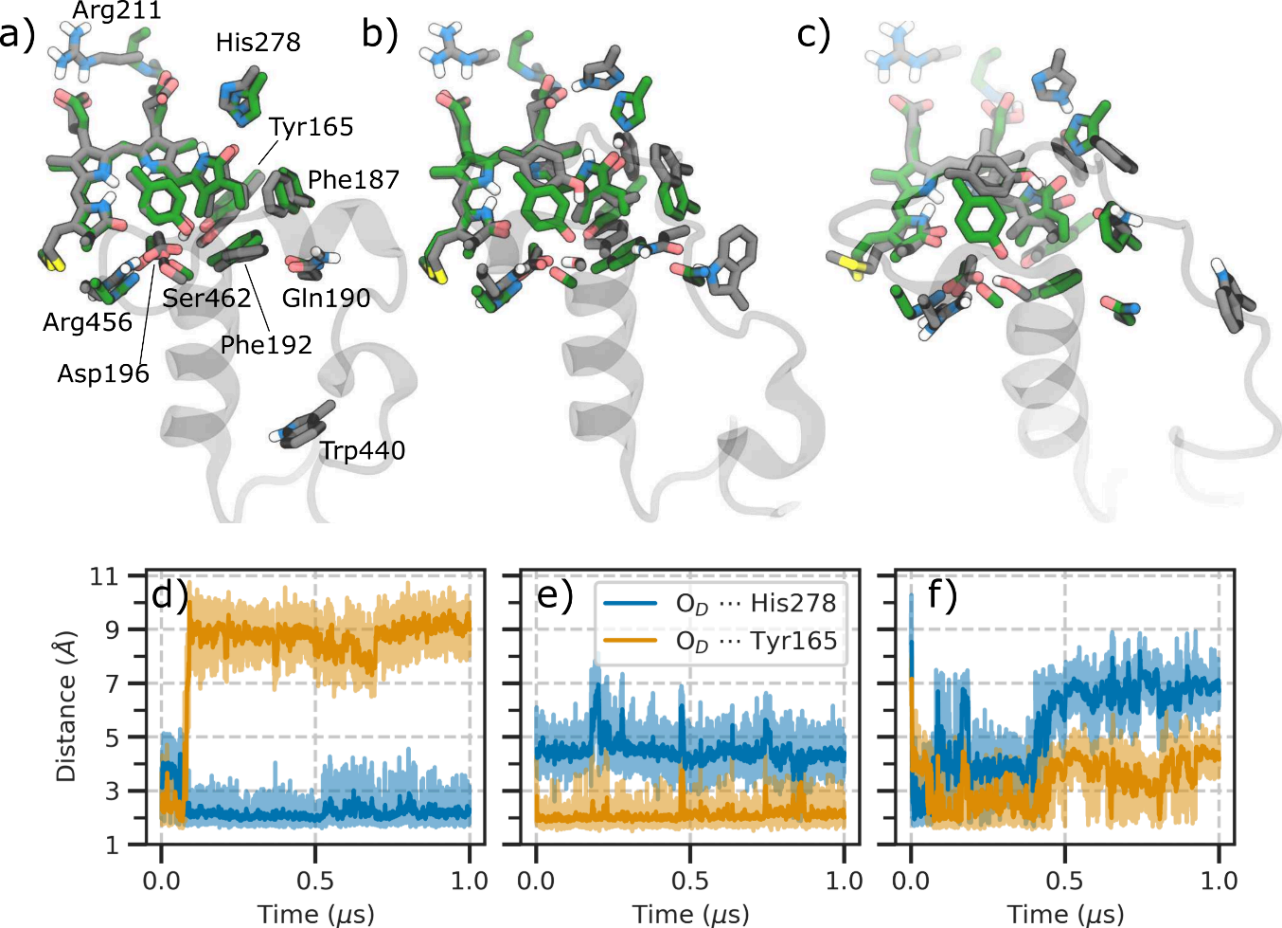
Evolution of Lumi-F. a), b), and c) Superimposition of the BV chromophore and the nearby residues in the BV–Pr (with rearrangements), BV–Pr (without rearrangements), and BV–Pr′ scenarios (gray), respectively. The experimental Meta-F intermediate (PDB ID: 6G20(24)) is shown in green. d), e), and f) Hydrogen bonding interactions between the D-ring carbonyl and either His278 or Tyr165 residues along three different MM MDs shown in a), b), and c), respectively.

To enhance the completeness of the sampling, we employed the Gaussian Accelerated Molecular Dynamics (GaMD) method,^48^ an unconstrained enhanced sampling technique. We run two independent 2 μs-long GaMD replicas starting from the BV–Pr after the rearrangements of the pocket (MD 1) and BV–Pr′ (MD 2). More details are reported in the Supporting Information.

In MD 1, BV remains stable in its Pr-like structure (Figure 6b, Figure S20b). Furthermore, we observe the cleavage of the interaction between Arg456 and Asp196, which is critical for the refolding of the tongue needed to reach the final Pr state. In MD 2 simulation started from the BV–Pr′, BV sporadically adopts a structure resembling the Pr-like one (Figure S20b). However, it consistently reverts to its original structure, as this transient state fails to interact with the His278 residue (Figure S20a) which is expected to trigger the changes of the binding pocket seen for the BV–Pr form (Figure 5a,d). Finally, only the rearrangements detected in the binding pocket of MD 1 spread throughout the entire apoprotein, resulting in a more significant tilt in the spine than in MD2 (Figure 6a). It is worth noting here that time-resolved X-ray solution scattering experiments^49^ suggest that another bathy phytochrome (PaBph) undergoes a large conformational change from the nearly parallel spine geometry in the Pfr state to the final O-framed structure in the active (Pr) state. To confirm that the observed conformational changes in the protein matrix are due to the photoisomerization and the associated changes in chromophore–protein interactions and not only to a more complete sampling, we performed a 1 μs-long GaMD simulation also on the dark state Pfr. Throughout the GaMD, the parallel spine geometry does not change (Figure S21) confirming that the tilt is due to the rearrangement of the residues following BV isomerization.

**Figure 6:**
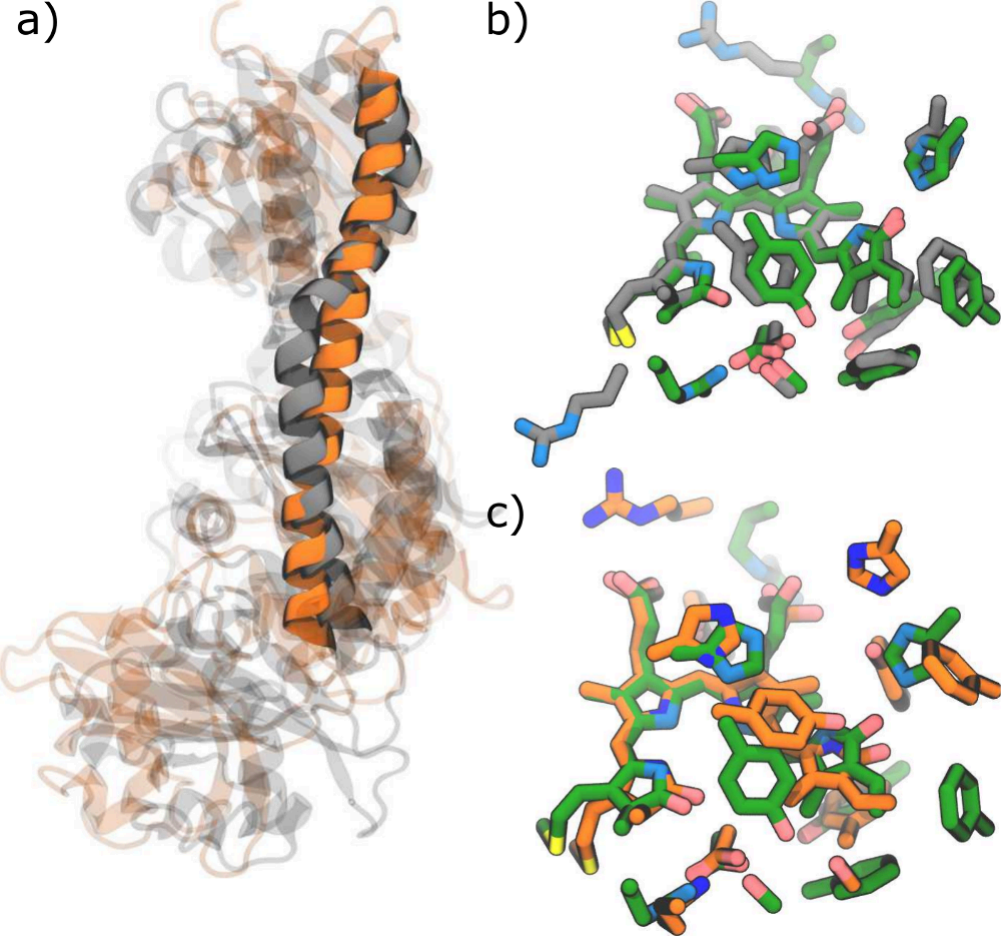
Comparison with the experimental Meta-F. a) Superimposition of two representative structures extracted from the two GaMD MDs started from BV–Pr (MD 1 in gray) and BV–Pr′ (MD 2, in orange). b) and c) Superimposition of the BV chromophore and the nearby residues in MD 1 (gray)/MD 2 (orange) vs the experimental Meta-F intermediate (PDB ID: 6G20(24)) (green).

In conclusion, our simulations reveal that the photoisomerization of the bathy Agp2 does not need a proton-coupled mechanism which would involve a too high energy path. Instead, the photoisomerization is reached with a 28% yield following the same mechanism observed for the canonical DrBph, i.e., through a hula-twist motion of the D5 and D6 dihedral angles of the excited BV which causes a counterclockwise rotation of the D ring.

Successively, the photoproduct relaxes in a first intermediate exhibiting a BV already in a structure similar to that found in the final (Pr) state. This contrasts with what found for the photoactivation of DrBph, where the first intermediate is characterized by a BV structure intermediate between those observed in the initial (Pr) and final (Pfr) states.^32^ The BV–Pr intermediate found in Agp2 is stabilized by hydrogen bond interactions between D-ring carbonyl and either Tyr165 or His278 residues and it can be interpreted as the Lumi-F intermediate, in agreement with a temperature scan cryo-crystallography experiment.^47^ Our findings, however, also indicate a second scenario (BV–Pr′) where BV does not present the Pr-like structure. This heterogeneity of Agp2 is here attributed to the dynamic nature of the protonated propionic group linked the C-ring; in fact, the second scenario rapidly coverts into the first one if we deprotonate Cprop. In DrBph, this heterogeneity was not found^50^ as Cprop is deprotonated and stabilized by strong hydrogen bonding interactions with the protein, resulting in a more rigid structure. To further support this explanation, we note that previous MD simulations performed by us on DrBph with a protonated BV^51^ revealed the presence of two basins similar to those found in this work, which were inaccessible when Cprop was deprotonated.^32^ Finally, exploring longer time scales we obtained that only the BV–Pr scenario generates a binding pocket similar to that experimentally observed for Meta-F^24^ (Figure 6b,c).

Combining all these findings we can identify analogies and differences in the photoactivation mechanism of the two phytochromes. Following the excitation, BV undergoes a photoisomerization through a hula-twist mechanism in both phytochromes. In Agp2, the photoproduct evolves into a first intermediate that is significantly more heterogeneous and dynamic compared to DrBph. We propose that this heterogeneity is resolved by a proton transfer from Cprop to His278, facilitating the structural convergence of BV into the fully rotated structure which characterizes the final Pr state. From there, local changes propagate to the binding pocket, causing a reorientation of some important residues, namely Tyr165, Phe187, and Phe192 (Figure 6b), up to the entire protein, resulting in a tilt of the spine. In DrBph instead the D-ring rotation remains incomplete in the first (Lumi-R) intermediate. This slows down the rearrangements of the residues in the binding pocket that remain limited up to the Meta-F.

## Supporting Information Available

Comprehensive overview of the multiscale strategy used to model the photoactivation mechanism of Agp2 along with all parameters used in the different simulations; study of the proton transfer mechanism through both a static (QM/MM optimizations) and dynamic (excited-state dynamics coupled with enhanced sampling technique) pathway; supporting figures (PDF)
Transparent Peer Review report available (PDF)
