# Supplementary material for: Analogies and Differences in the Photoactivation Mechanism of Bathy and Canonical Bacteriophytochromes Revealed by Multiscale Modeling
Source: J Phys Chem Lett. 2024 Aug 1;15(31):8078–84. doi: 10.1021/acs.jpclett.4c01823 (PMC11376688; doi:10.1021/acs.jpclett.4c01823)
Supplement: Supplementary file 1 — jz4c01823_si_001.pdf [file jz4c01823_si_001.pdf]

Supplementary Information: Analogies and Differences in the Photoactivation Mechanism of Bathy and Canonical Bacteriophytochromes Revealed by Multiscale Modeling

Giacomo Salvadori[1,*] and Benedetta Mennucci[2,*]

[1]Institute for Computational Biomedicine (INM-9/IAS-5), Forschungszentrum Jülich, 52428, Jülich, Germany

[2]Dipartimento di Chimica e Chimica Industriale, University of Pisa, via G. Moruzzi 13, 56124, Pisa, Italy

[*]Corresponding Author, e-mail: g.salvadori@fz-juelich.de benedetta.mennucci@unipi.it

S1 Methods

A multiscale strategy has been applied for modeling the photoactivation mechanism of Agp2 (Fig. S1). First, we sampled the configurational space of the solvated dimeric system in the Pfr state through four μs-long MM MDs. Subsequently, after a refinement of the PES of the QM subsystem achieved with ps-long QM/MM MDs, we investigated the photochemical process with the surface hopping method.[1,2] Then, to investigate the evolution of the photoproduct and to characterize the first (Lumi-F) intermediate, we used ground-state (QM/)MM MDs. Finally, we tried to reach the second (Meta-F) intermediate by using the Gaussian Accelerated Molecular Dynamics (GaMD) method.

To investigate the possibility of an ultrafast proton transfer, we tested both static and dynamic models. In the former, we performed both ground- and excited-state QM/MM optimizations, while in the latter we combined enhanced sampling techniques (OPES EXPLORE) with excited-state polarizable QM/MM MDs.

Sampling the Pfr state

The initial structural model of the Agp2 phytochrome in the resting (Pfr) state was generated starting from the 6G1Y[3] entry of the Protein Data Bank. MODELLER[4] was used to add the missing residues in the crystal structure, namely Met1, Ala2, Ser3, Thr4, Asp5, Thr81, Gly82, Arg83, and Thr84 for both chains A and B, Ser121 and Asp122 for chain A, and Thr85 for chain B. The structure was subjected to further loop refinement to improve global structure quality using

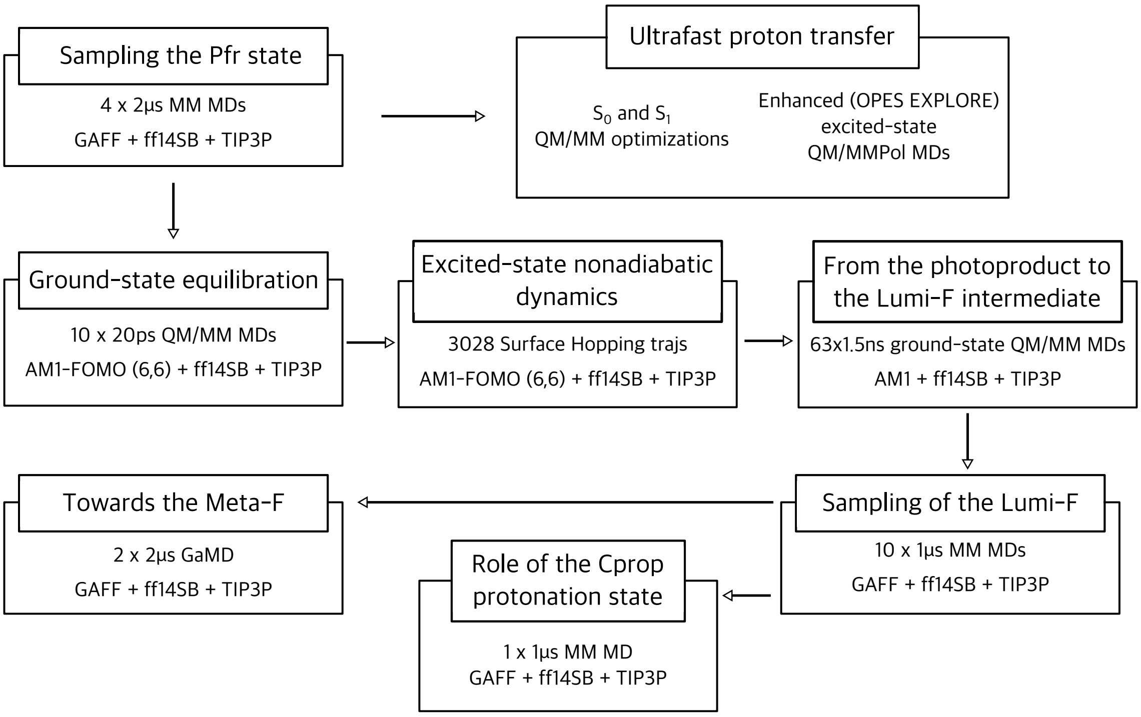

Figure S1: Multiscale strategy applied for modeling the photoactivation mechanism of Agp2.

the ModRefiner web server.[5,6] H++ web server was used to determine the protonation at pH 7. In particular, His248 and His278 were protonated on the Nϵ site.[7–9] We modeled the biliverdin chromophore (BV) with all pyrrole rings and the ring C propionic side chain protonated.[8–11] Partial charges of the BV were computed using the RESP protocol[12] on top of a B3LYP/6-311G(d,p) electrostatic potential calculation.

The chromophore and its bonding interactions with the covalently bonded cysteine residue (Cys13), were described with the GAFF force-field[13]. Our GAFF parametrization of BV was based on a careful choice of atom types to conform to the conjugation pattern of the chromophore. This force field for BV was already used in previous work on both the (resting) Pr and (active) Pfr states of the DrBph phytochrome;[14,15] the resulting ensembles successfully reproduced different spectroscopic properties of the system, indicating that GAFF (with our assignment of parameters) can indeed properly describe BV. The protein was described with the AMBER ff14SB forcefield,[16] while water molecules with the TIP3P model.[17] The refined dimeric system (protein and crystallographic waters) was first subjected to an *in vacuo* minimization with a 10 kcal mol^{-1}Å^{-1} harmonic restraint on backbone atoms; then, the entire dimer was soaked in a truncated octahedron water box and electrically neutralized with 0.15M NaCl.[18] The solvated system was subjected to two minimization cycles of 5000 steps each: 2500 steps using steepest descent and other 2500 steps using conjugate gradient. In the first cycle, we restrained backbone atoms with a 10 kcal mol^{-1}Å^{-1} harmonic potential. Then, the entire system was minimized without constraints. A 1 ns-long heating to 300 K in an NVT ensemble followed, restraining the movement of the chromophores, the protein backbone, and the crystal water oxygens with a 10 kcal mol^{-1}Å^{-2} harmonic potential. To assure a slow release of the restraints, we performed three different 2 ns-long NPT equilibration runs by

gradually decreasing the restraints to 1 kcal $mol^{-1}Å^{-2}$.

The production run was carried out without any restraint for 2 μs in the NPT ensemble, of which we discarded the first 500 ns. Average-quantity analyses were made on the remaining 1.5 μs (15000 frames per trajectory).

All simulations were performed applying the particle mesh Ewald (PME) truncation method (with a short-range cut-off of 10 Å), an integration step of 2 fs, the SHAKE algorithm, a Langevin thermostat with a collision frequency of 1 ps^{-1}, and the Monte Carlo barostat for NPT simulations. All molecular dynamics simulations were run with AMBER18.[19–23]

We performed a clustering analysis on 12000 structures extracted from the four MM MD replicas based on hydrogen bond donor-acceptor distances involving the D ring and the nearby residues (Fig.S2). The final dataset comprises 12000 structures per 8 distances.

To reduce the dimensionality of the problem, we have used a principal component analysis (PCA) based on intermolecular distances involving key residues in the chromophore-binding pocket. On top of that, we have applied a hierarchical clustering algorithm finally obtaining three clusters. In cluster 1, the D-ring carbonyl does not interact with a water molecule, but instead with Gln190. In both clusters 0 and 2, the D-ring carbonyl interacts with (at least) one water molecule, but in cluster 0 there is no hydrogen bond between the Cprop group and water molecules (Fig. S2).

Nonadiabatic dynamics

We extracted ten representative configurations from the cluster analysis performed on the Pfr state (see Fig. S2) and used them as starting points for ground-state QM/MM-MD trajectories. Each trajectory was run for 20 ps using a timestep Δt=0.5 fs and the Bussi-Parrinello stochastic thermostat at 300K. . We reduced the original dimeric system by keeping only the residues (protein and water molecules) within 26 Å from the chromophore to keep the computational cost feasible. The part of protein and solvent at more than 22 Å away from the BV were kept frozen. The BV chromophore was treated with the multi-reference floating occupation molecular orbital-complete active space configuration interaction (FOMO-CASCI) method[24,25] in combination with the AM1 Hamiltonian.[26]We used an active space of (6,6) combined with a gaussian width of 0.05 a.u. for FOMOs, as it has already been used and validated in previous work.[27] The boundary between the covalently linked QM and MM parts was treated using a connection atom scheme previously used for semiempirical QM/MM approaches.[28] According to this scheme, the $C_{3\beta}$ carbon atom of the chromophore is the connection atom which behaves as a hydrogen atom in the QM part and as a normal carbon atom in the MM part. Conversely, the protein was described with the AMBER ff14SB force field and water molecules with TIP3P, as in the fully MM MDs. The QM/MM trajectories were performed with a modified version of the semiempirical program MOPAC2002,[29] interfaced with the TINKER software package.[30]

The last 15 ps of the ten ground-state QM/MM trajectories were used to extract the initial conditions (nuclear coordinates and momenta) for generating the swarm of non-adiabatic surface hopping trajectories. This sampling has been performed by taking into account both the excitation energy and the radiative transition probability (Fig. S3, S4).[25]. Since the oscillator strength for the S_0-to-S_2 excitation is small and the first excited state is well separated from the second one, all the

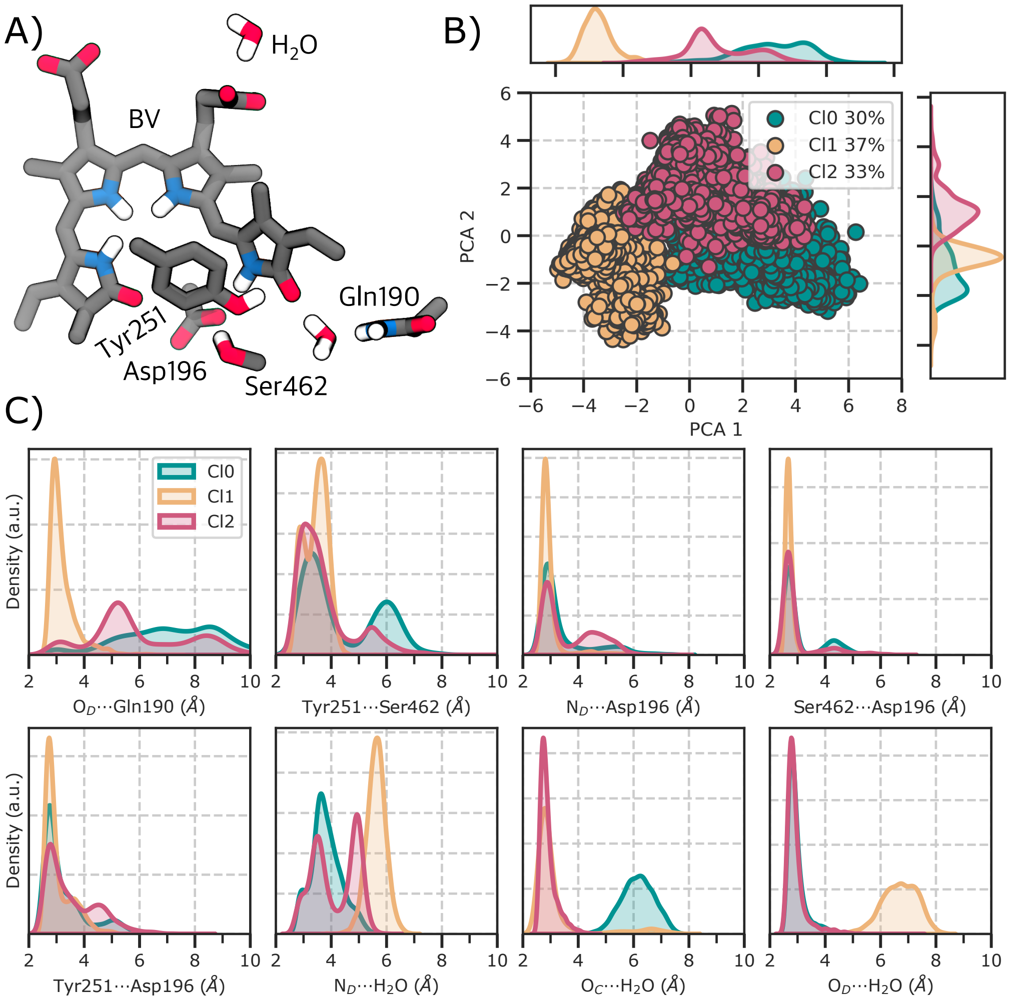

Figure S2: A) Representation of the BV chromophore with their nearby residues. B) Clustering visualized in the PCA space. Points differently colored correspond to different clusters obtained with an agglomerative clustering: cluster 0 (Cl0) in green, cluster 1 (Cl1) in yellow, and cluster 2 (Cl2) in red). The density distribution is shown for both PCA 1 and PCA 2. C) KDE distribution of the distances used in the PCA analysis that separate the three clusters.

trajectories have been started in the first singlet excited state.

In all SH trajectories, the QM and the MM subsystems and their respective levels of theory were the same used in the ground-state QM/MM simulations. The local diabatization algorithm[24,25] was used for the integration of the time-dependent Schrödinger equation for the electrons, the timestep Δt was reduced to 0.1 fs, and quantum decoherence was taken into account by applying an energy-based correction.[31] For all non-adiabatic trajectories an energy conservation threshold of c.a. 5 kcal mol^{-1} was set. After a backward hop, we rescale the component of the velocity vector parallel to the non-adiabatic coupling vector between the old and the new active states. Regarding forward hops, the nuclear velocity vector is rescaled by keeping its direction unaltered. We forbid hops when the energy gap between electronic states S_0 and S_1 was larger than 0.5 eV. To reduce the computational cost, trajectories were stopped when one of the following two criteria

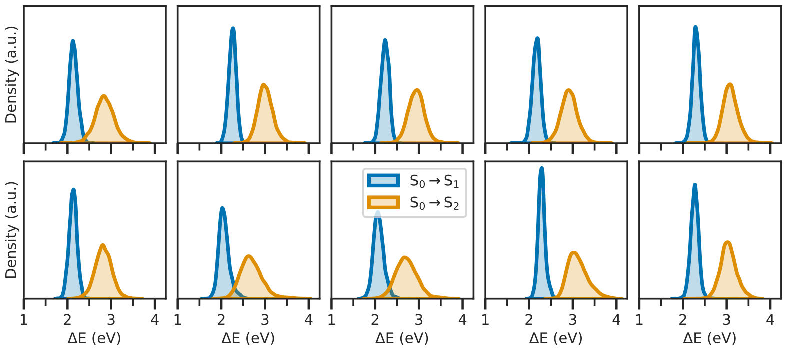

Figure S3: Distribution of the energy difference between S_0 and S_1 and between S_0 and S_2 for each replica.

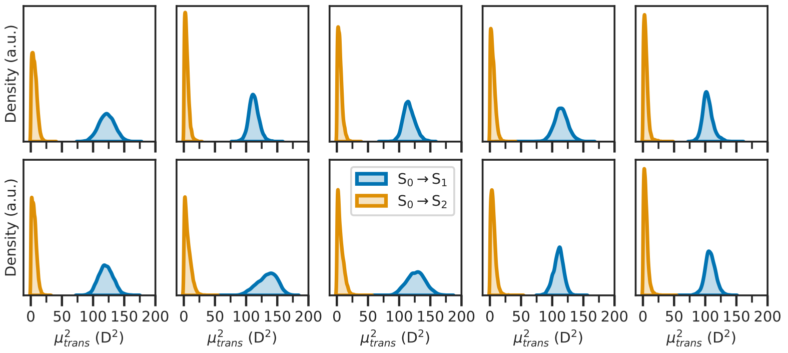

Figure S4: Distribution of the squared transition dipole moment between S_0 and S_1 and between S_0 and S_2 for each replica.

was fulfilled: (i) the trajectory has been running on the ground state surface for 1 ps; (ii) the total simulation time is larger than 20 ps.

Surface Hopping simulations were performed with a modified version of the semiempirical program MOPAC2002,[29] interfaced with the TINKER software package.[30]

All the figures, the analysis of the trajectories, and the fitting were performed with matplotlib[32], in-house FORTRAN 90 scripts, SciPy[33], and in-house Python scripts.

From the protoproduct to the Lumi-F

Representative configurations of the photoproduct obtained from the last frames of the reactive SH trajectories, i.e., 1 ps after the $S_1 \rightarrow S_0$ hop, were used to propagate a swarm of ground-state MM MD simulations in search of the Lumi-F intermediate. In these simulations, we reintroduced a full solvation of the protein using a truncated octahedron water box. Both QM/MM and MM MD simulations were performed. In the former case, an HF/AM1 approach has been used to describe the chromophore to avoid discontinuity with respect to the nonadiabatic SH simulations.

The solvatated system was subjected to 400 steps of energy minimization using steepest descent and other 600 steps using conjugate gradient. Then, a gradually heating to 300 K in a NVT ensemble

in 20 ps followed, restraining the movement of the chromophore and the protein backbone with a 4 kcal $mol^{-1}Å^{-2}$ harmonic potential. It followed a 30 ps NPT equilibration run with the same restraints on the chromophore and all the protein backbone. Sixty-three QM/MM trajectories were propagated in the NPT ensemble for 1.5 ns each and average-quantity analyses were made on 15000 frames per trajectory.

Sampling the Lumi-F

Finally, to extend the investigation of the intermediate evolution in the μs-timescale, we switched to a fully MM description using the same FF used for the sampling of the Pfr state. Ten independent replicas were run for 1 μs each and average-quantity analysis were made on 10000 frames per trajectory.

The solvated system was subjected to 400 steps of energy minimization using steepest descent and other 1600 steps using conjugate gradient. Then, a gradually heating to 300 K in a NVT ensemble in 400 ps followed, restraining the movement of the chromophore and the protein backbone with a 4 kcal $mol^{-1}Å^{-2}$ harmonic potential. The same constraints were maintained for the following 1 ns-long equilibration step. The production run was carried out without any restraint for 1 μs in the NPT ensemble.

For both QM/MM and MM MDs, the protein and water were described with the same force field used in the excited state simulations.

All simulations were performed applying the particle mesh Ewald (PME) truncation method (with a short-range cut-off of 10Å), an integration step of 2 fs, the SHAKE algorithm, a Langevin thermostat with a friction coefficient of 1 ps^{-1}, and the Monte Carlo barostat for NPT simulations. All molecular dynamics simulations were performed with AMBER22[34,35].

Towards the Meta-F

The parameters used for the Gaussian accelerated MD (GaMD)[36] were the same as those used for the unbiased MM MD. We have used a dual-boost protocol on both dihedral ($\sigma_D = 6.0$ kcal mol^{-1}) and nonbonded potential energy of the system ($\sigma_P = 6.0$ kcal mol^{-1}) and set the system threshold energy to the lower bound $E = V_{max}$. All GaMD simulations followed the following protocol. First, we ran a 4 ns-long conventional MD without any boost potential. During the first 0.8 ns, no statistics were collected, i.e., the maximum (V_{max}), minimum (V_{min}), average (V_{avg}), and standard deviation (σ_V) of the system potential, while in the remaining 3.2 ns potential statistics are collected. Then, a 100 ns-long GaMD equilibration was conducted: during the first 3.2 ns, boost parameters were not updated, while in the remaining 96.8 ns, they were updated. Following this, a 2 μs-long GaMD production run was conducted where the boost was applied, and the parameters not updated.

S2 The proton transfer mechanism

QM/MM optimizations

To investigate the hypothesis of a deprotonation of the NH group of D ring via the carbonyl group, we first performed both ground- and excited-state QM/MM optimizations of BV before (keto) and after (enol) the proton transfer from nitrogen to oxygen. We used six different configurations of the system, extracted from our clusters of the Pfr state, that differ for the presence/absence of water molecules close to the D-ring carbonyl (Fig. S8). We selected three configurations with one (or more) water molecules coordinated to the D-ring, and three configurations without such an interaction.

Since the process is expected to complete in 50 fs,[9] all the calculations were performed assuming that the protein is frozen in the initial configuration before the proton transfer. The QM subsystem is comprehensive of the BV chromophore, the sidechain of the covalently-bound cysteine residue, and an aspartate residue (Asp196), and all the water molecules within 3Å from the D-ring oxygen/nitrogen. The ground-state optimizations have been performed at ωB97XD/6-31G(d,p) level of theory whereas, to better describe the excited states, the basis set has been enlarged to include diffusion functions (6-31+G(d,p)). The frequency calculations to obtain the zero-point correction were computed at the same level of theory. All the calculations were performed with Gaussian16.[37]

The energy differences between the keto-like and enol-like forms obtained for all the selected configurations are shown in Tab. S1.

Structures	Ground state	Excited state
I	39.0	37.7
II	34.2	33.1
III	31.5	33.5
IV	26.2	26.5
V	27.5	29.4
VI	26.6	27.2
I_{Asp}	46.3	47.4
IV_{Asp}	24.4	26.1

Table S1: Energy difference (enol − keto) in the ground and excited states in kcal mol^{-1}. Structures I to III are characterized by the absence of water molecules within hydrogen bonding distance (3.2Å) of the D-ring oxygen/nitrogen, while structures IV to VI have at least one water molecule within 3.2Å of the same atoms. In structures I_{Asp} and IV_{Asp}, we used the same structures as in I and IV to simulate proton transfer to Asp196.

QM/MM MD simulations

Overall, we ran ten excited-state polarizable embedding QM/MM[38] MD simulations starting from 10 frames extracted from the three clusters of the plain MM MD simulations on the Pfr state. For each snapshot, we kept a shell containing only one monomer and solvent residues within 10 Å of

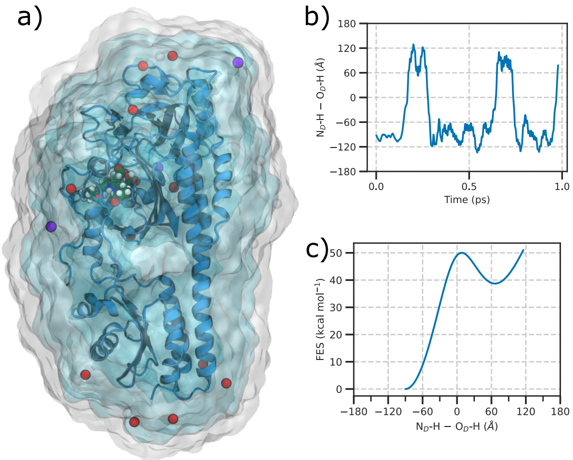

Figure S5: a) Depiction of the reduced system used for the QM/MMPol MD simulations. In gray are represented all the frozen residues; in red and violet, the sodium and chloride ions, respectively; in green, the BV chromophore. b) Trend of the collective variable used in one biased QM/MMPol simulation with the respective free energy (c).

the protein. Non-periodic boundary conditions were included by freezing all the residues beyond 6 Å from the protein (Fig. S5a). The BV chromophore and the sidechain of the covalently-bound cysteine residue were treated at the ωB97XD/6-31G(d,p) level of theory, whereas the remaining part of the system was described using the AMOEBA-BIO-09 force field.[39] The latter employs fixed charges, dipoles, and quadrupoles to describe the electrostatics and an induced-point-dipole (IPD) model to describe polarization.[40,41] To generate suitable starting conditions for the MD simulations, on each of the structures, we first performed a QM/AMOEBA geometry optimization using the limited-memory Broyden-Fletcher-Goldfarb-Shannon algorithm[42] and then an 8 ps-long ground-state equilibration in the NVT ensemble using an integration step of 0.5 fs and the Velocity Verlet algorithm. A constant temperature of 300 K was kept using the Bussi thermostat[43] with a time constant of 0.1 ps. For all trajectories, a 12.0 Å cutoff was applied for the Van der Waals interactions. All QM/AMOEBA simulations were carried out using an interface[44–46] between the MD package Tinker[47,48] and a locally modified version of the Gaussian suite of programs.[49]

From these ground-state simulations, we extracted the ten structures for the excited-state QM/AMOEBA MD simulations in the NVT ensemble. To accelerate the sampling, we profit from the flexibility and efficiency of the On-the-fly Probability Enhanced Sampling (OPES) method in its EXPLORE flavour.[50,51] The OPES parameters used were 45 kcal mol^{-1} for the energy barrier, and 50 steps for the MD pace (25 fs). The number of MD steps for measuring adaptive sigma was set to 150. The biased simulations have been carried out using the Gaussian-Tinker-Plumed (Plumed v2.8.2) machinery.[52–54] The collective variable (CV) chosen to represent the process is the difference in the proton distance from the D-ring nitrogen and oxygen. The trend of the CV for all the trajectories is shown in Fig.S6.

The obtained results fully confirm what was found with the static picture, namely an extremely unfavorable proton transfer process (Fig.S5c). As a matter of fact, some trajectories did not even show a transition to the enol-like state, despite the use of a very high BARRIER parameter in the OPES EXPLORE simulations.

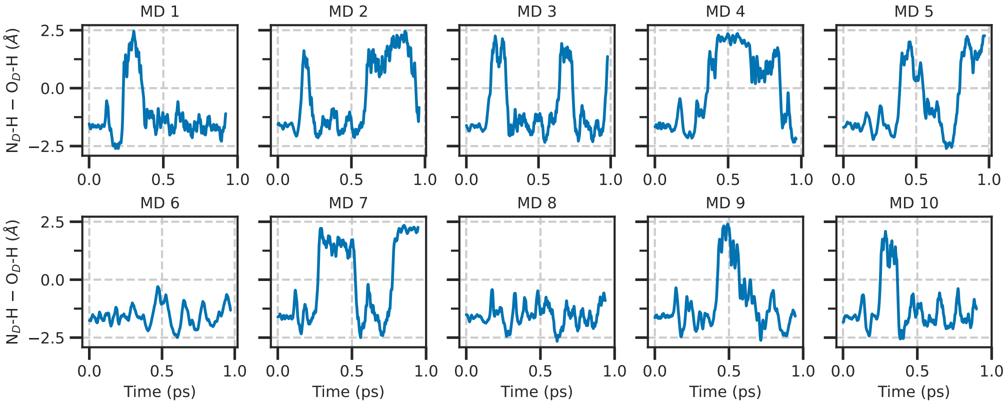

Figure S6: Trend of the collective variable in all QM/MMPol biased simulations

S3 Supplementary Figures

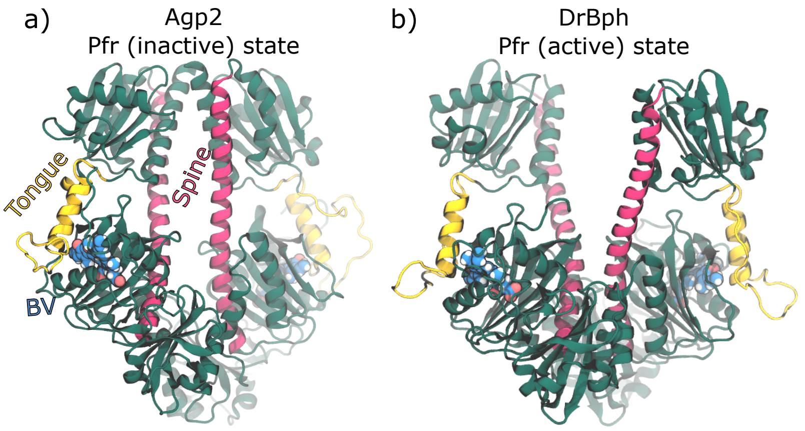

Figure S7: a), b) Crystallographic structures of the Pfr state of Agp2 (PDB ID: 6G1Y [3]) and DrBph (PDB ID: 5C5K [55]). The tongue and the spine are highlighted in yellow and red, respectively.

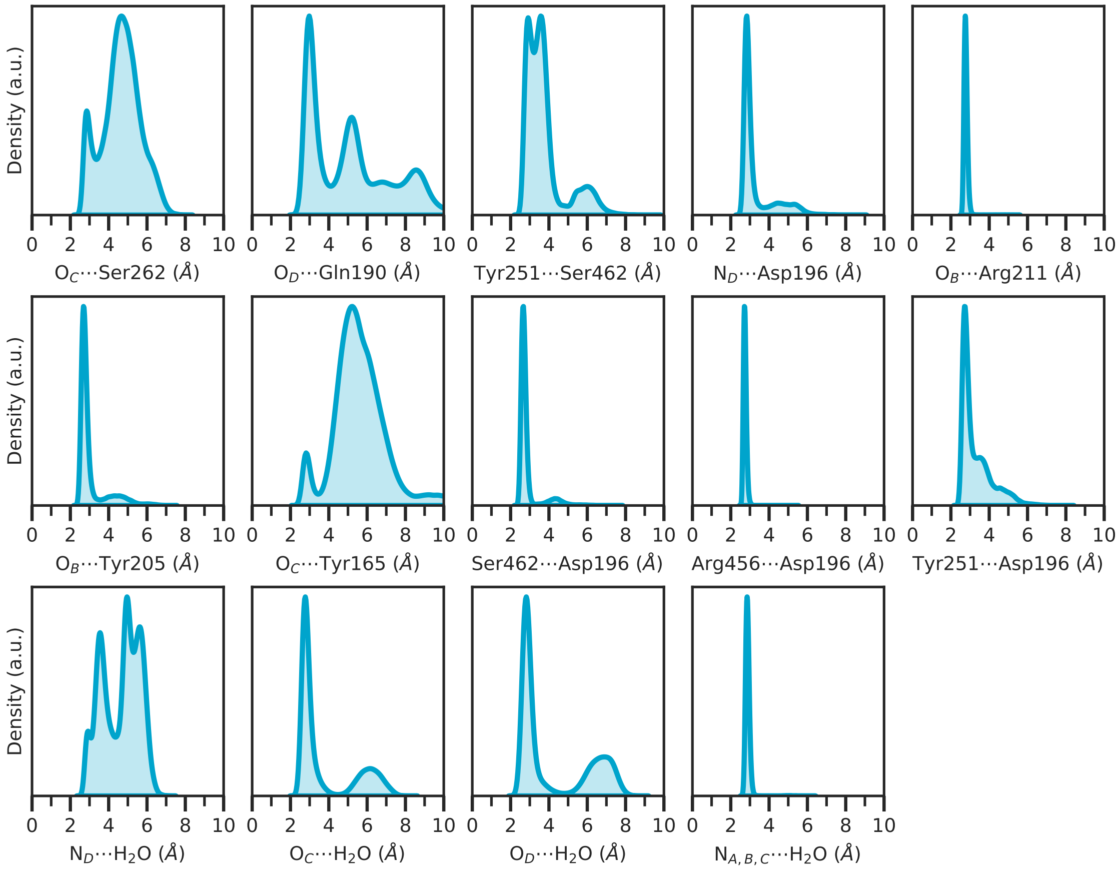

Figure S8: Distributions of the main chromophore-protein interactions along the four Pfr MM MDs.

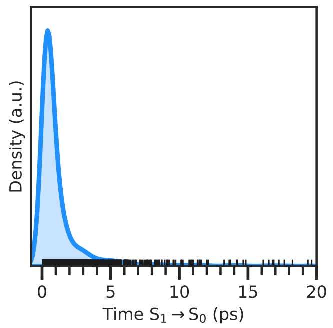

Figure S9: KDE distribution of the S_1 $\rightarrow S_0$ hopping times for all trajectories (both reactive and nonreactive). Each black stick represents a hop event.

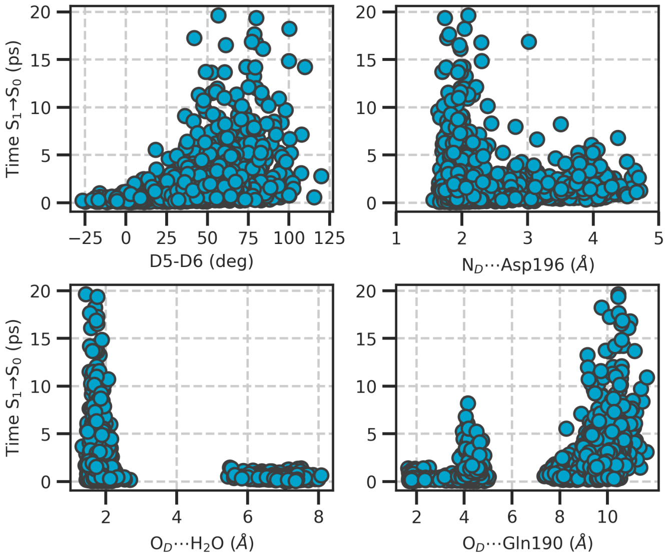

Figure S10: Scatter plots of the D5 - D6 dihedrals difference, $N_D \cdots$Asp196, $O_D \cdots H_2O$, and $O_D \cdots$Gln190 interactions against the hopping S_1-to-S_0 times. The distances are all minimum hydrogen bonding distances.

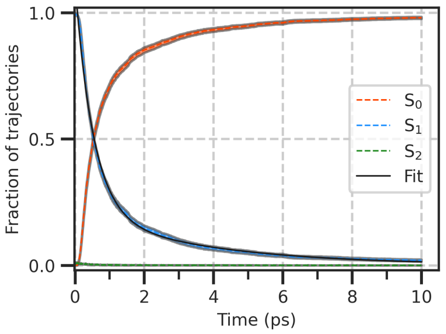

Figure S11: Time evolution of the electronic state populations, evaluated as the fraction of trajectories running in the given state at the given time, with their 95 % confidence intervals. Black line: fit of the S_1 population according to the following biexponential function $A_1 \exp -t/t_1 + A_2 \exp -t/t_2$. $A_1 = 0.34$, $A_2 = 0.74$, $t_1 = 4.25$, $t_2 = 0.71$.

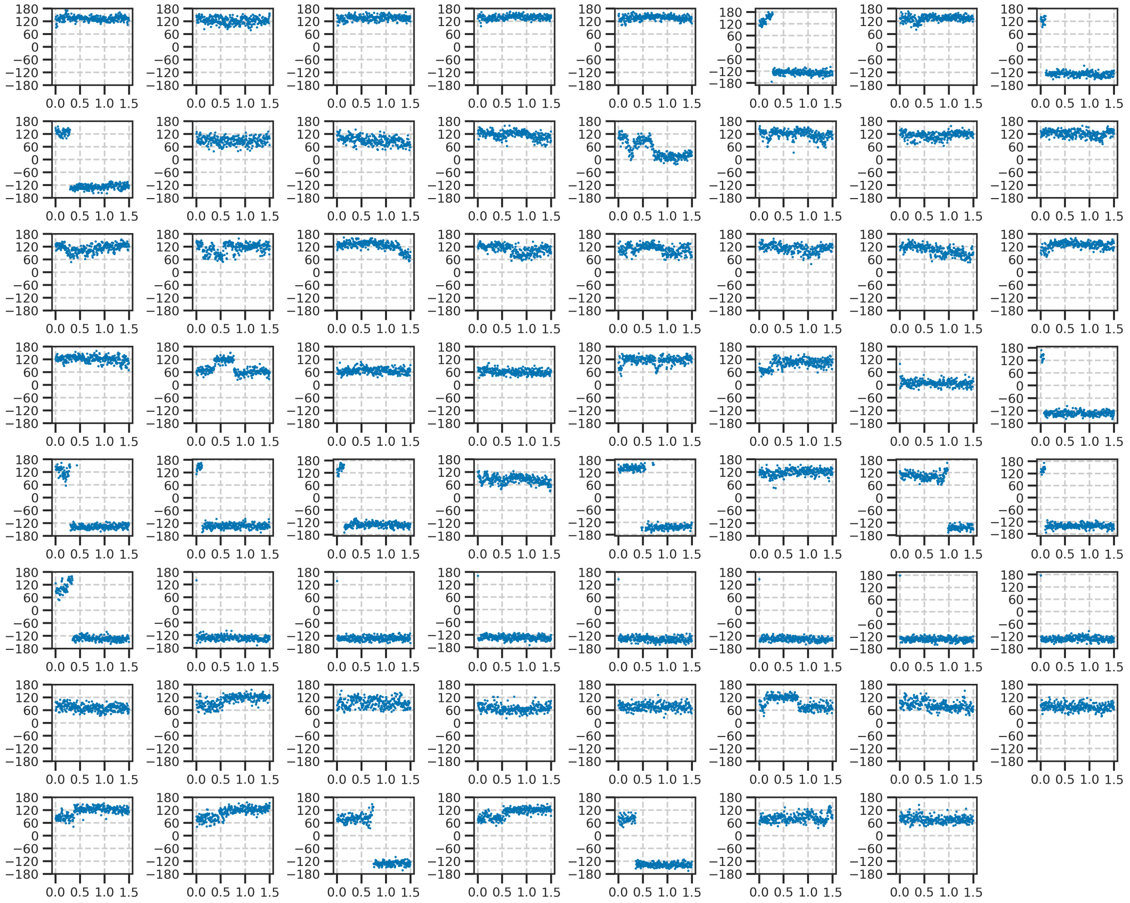

Figure S12: Trend of the dihedral D5 for each QM/MM MDs.

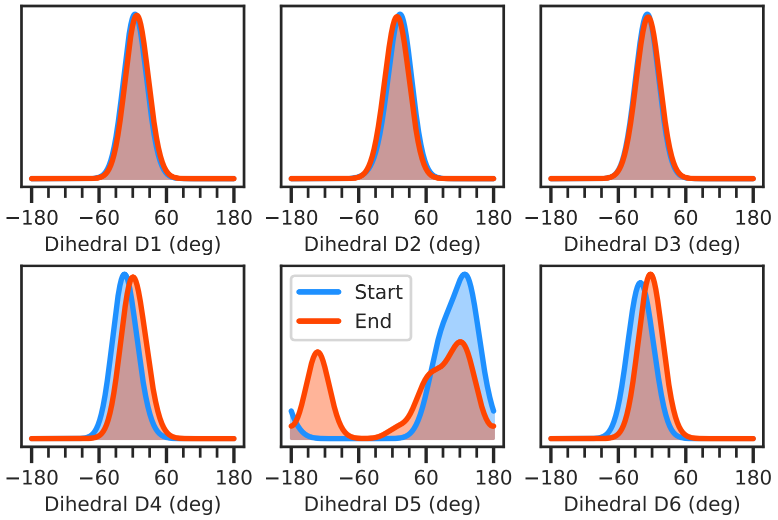

Figure S13: KDE distributions for the dihedrals D1−D6. In black, blue, and orange are represented the distributions of the Pfr state, after the SH simulations, and after 1.5 ns-long QM/MM MDs, respectively.

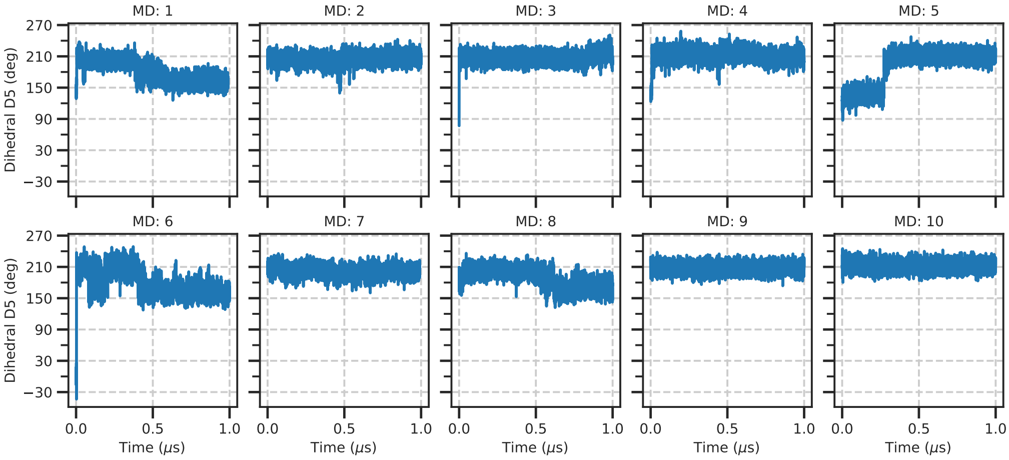

Figure S14: Trend of the dihedral D5 for each MM MDs.

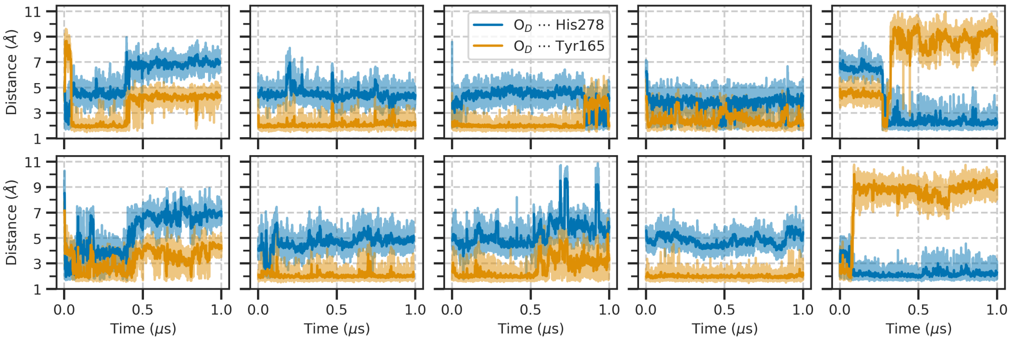

Figure S15: HB distance between the D-ring carbonyl and the His278 or Tyr165 residues. In bold, a moving average with a window of 20 frames.

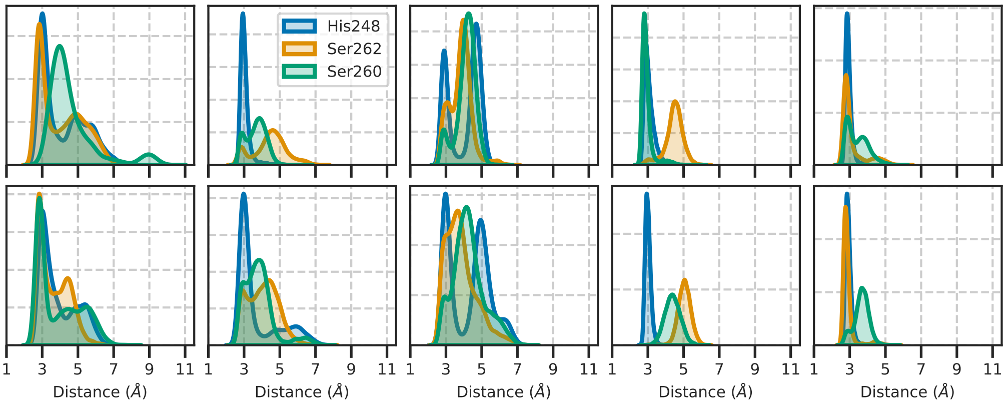

Figure S16: KDE distribution for the $O_C \cdots$His248,Ser262, and Ser260 distances. Each box represents a different trajectory. The distance is the minimum distance between hydrogen bonding heavy atoms.

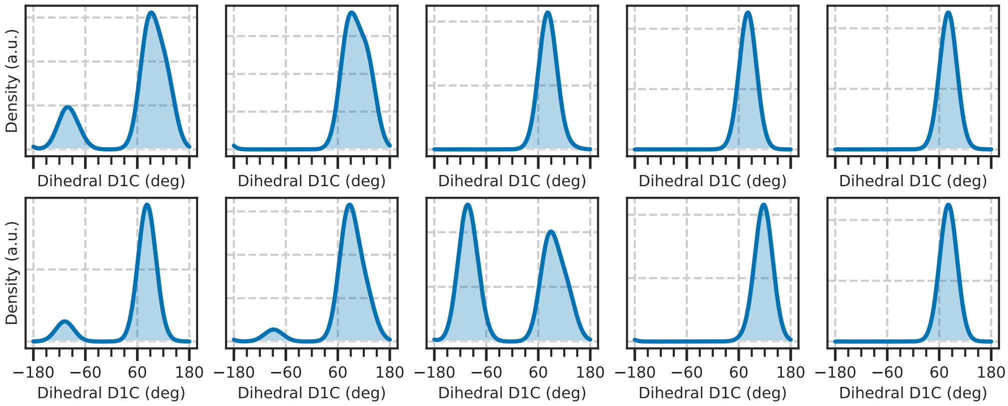

Figure S17: KDE distribution of the dihedral D1C. Each box represents a different trajectory.

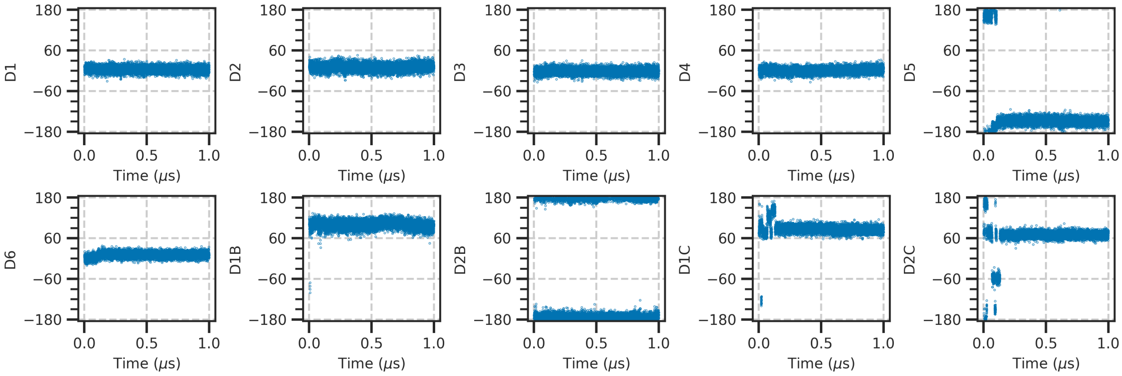

Figure S18: Trend of the main dihedrals computed along the MM MD with a deprotonated BV

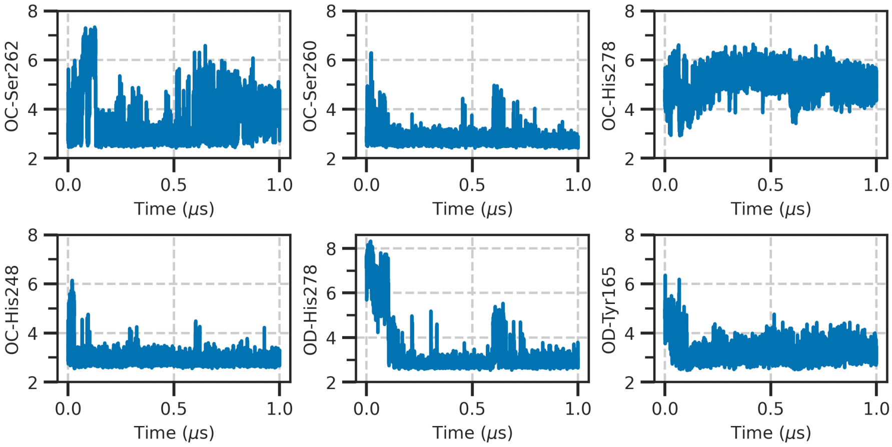

Figure S19: Trend of the main interactions between the C-ring propionate and the protein computed along the MM MD with a deprotonated BV

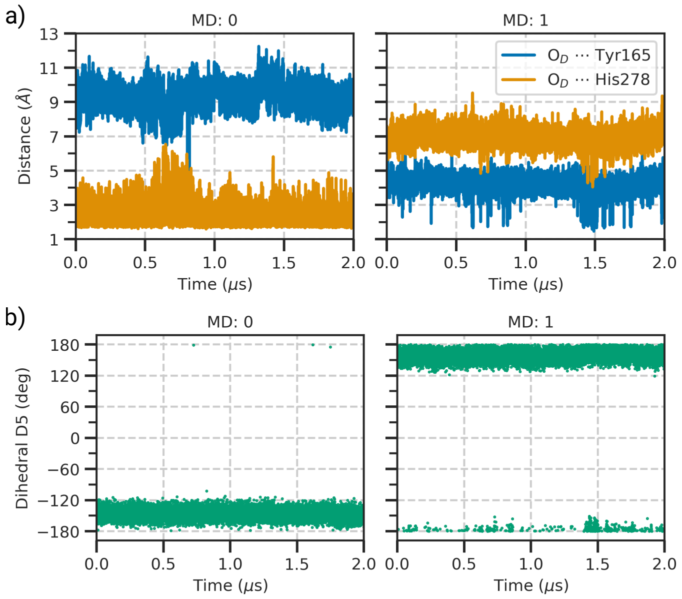

Figure S20: a) Trend of the hydrogen bonding distance between the carbonyl group and Tyr165 or His278 along the two GaMD MDs. b) Trend of the dihedral D5 along the two GaMD MDs.

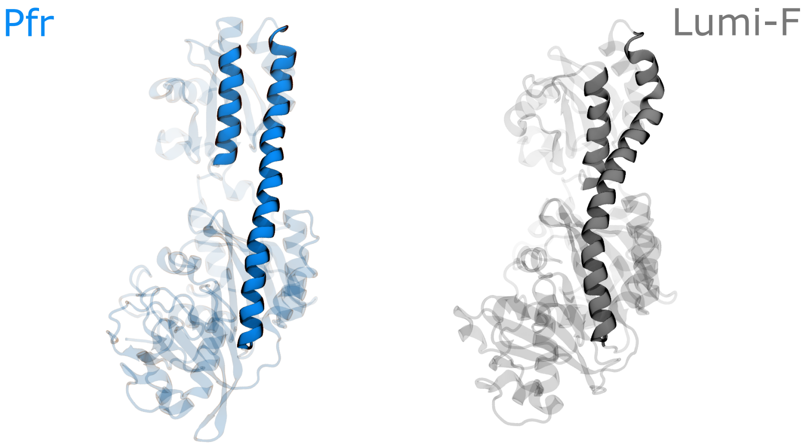

Figure S21: Comparison of the structures obtained after a GaMD on both the Pfr and Lumi-F states. The spine has been highlighted to better understand the tilt.

References

[1] Tully, J. C. Molecular dynamics with electronic transitions. *J. Chem. Phys* **93**, 1061–1071 (1990).

[2] Tully, J. C. Perspective: Nonadiabatic dynamics theory. *J. Chem. Phys.* **137**, 22A301 – 7 (2012).

[3] Schmidt, A. *et al.* Structural snapshot of a bacterial phytochrome in its functional intermediate state. *Nat. Commun.* **9**, 4912 (2018).

[4] Sánchez, R. & Šali, A. Evaluation of comparative protein structure modeling by MODELLER-3. *Proteins Struct. Funct. Genet.* **29**, 50–58 (1997).

[5] Fiser, A., Do, R. K. G. & Šali, A. Modeling of loops in protein structures. *Protein Sci.* **9**, 1753–1773 (2000).

[6] Fiser, A. & Sali, A. ModLoop: Automated modeling of loops in protein structures. *Bioinformatics* **19**, 2500–2501 (2003).

[7] Lopez, M. F. *et al.* Role of the propionic side chains for the photoconversion of bacterial phytochromes. *Biochemistry* **58**, 3504–3519 (2019).

[8] Kraskov, A. *et al.* Intramolecular Proton Transfer Controls Protein Structural Changes in Phytochrome. *Biochem.* **59**, 1023–1037 (2020).

[9] Yang, Y. *et al.* Ultrafast proton-coupled isomerization in the phototransformation of phytochrome. *Nat. Chem.* **14**, 823–830 (2022).

[10] Escobar, F. V. *et al.* A protonation-coupled feedback mechanism controls the signalling process in bathy phytochromes. *Nat. Chem.* **7**, 423–430 (2015).

[11] Escobar, F. V. *et al.* Common structural elements in the chromophore binding pocket of the pfr state of bathy phytochromes. *Photochem. Photobiol.* **93**, 724–732 (2017).

[12] Bayly, C. I., Cieplak, P., Cornell, W. D. & Kollman, P. A. A well-behaved electrostatic potential based method using charge restraints for deriving atomic charges: The RESP model. *J. Phys. Chem.* **97**, 10269–10280 (1993).

[13] Wang, J., Wolf, R. M., Caldwell, J. W., Kollman, P. A. & Case, D. A. Development and testing of a general amber force field. *J. Comput. Chem.* **25**, 1157–1174 (2004).

[14] Macaluso, V., Cupellini, L., Salvadori, G., Lipparini, F. & Mennucci, B. Elucidating the role of structural fluctuations, and intermolecular and vibronic interactions in the spectroscopic response of a bacteriophytochrome. *Phys. Chem. Chem. Phys.* **22**, 8585–8594 (2020).

[15] Macaluso, V., Salvadori, G., Cupellini, L. & Mennucci, B. The structural changes in the signaling mechanism of bacteriophytochromes in solution revealed by a multiscale computational investigation. *Chem. Sci.* **12**, 5555–5565 (2021).

[16] Maier, J. A. *et al.* ff14SB: Improving the Accuracy of Protein Side Chain and Backbone Parameters from ff99SB. *J. Chem. Theory Comput.* **11**, 3696–3713 (2015).

[17] Jorgensen, W. L., Chandrasekhar, J., Madura, J. D., Impey, R. W. & Klein, M. L. Comparison of simple potential functions for simulating liquid water. *J. Chem. Phys.* **79**, 926–935 (1983).

[18] Schmit, J. D., Kariyawasam, N. L., Needham, V. & Smith, P. E. SLTCAP: A Simple Method for Calculating the Number of Ions Needed for MD Simulation. *J. Chem. Theory Comput.* **14**, 1823–1827 (2018).

[19] Case, D. A. *et al.* Amber 2018 (2018). University of California, San Francisco.

[20] Lee, T.-S. *et al.* GPU-accelerated molecular dynamics and free energy methods in amber18: Performance enhancements and new features. *J. Chem. Inf. Model.* **58**, 2043–2050 (2018).

[21] Le Grand, S., Götz, A. W. & Walker, R. C. SPFP: Speed without compromise - A mixed precision model for GPU accelerated molecular dynamics simulations. *Comput. Phys. Commun.* **184**, 374–380 (2013).

[22] Götz, A. W. *et al.* Routine microsecond molecular dynamics simulations with AMBER on GPUs. 1. generalized born. *J. Chem. Theory Comput.* **8**, 1542–1555 (2012).

[23] Salomon-Ferrer, R., Götz, A. W., Poole, D., Le Grand, S. & Walker, R. C. Routine microsecond molecular dynamics simulations with AMBER on GPUs. 2. Explicit solvent particle mesh ewald. *J. Chem. Theory Comput.* **9**, 3878–3888 (2013).

[24] Granucci, G., Persico, M. & Toniolo, A. Direct semiclassical simulation of photochemical processes with semiempirical wave functions. *J. Chem. Phys.* **114**, 10608–10615 (2001).

[25] Persico, M. & Granucci, G. An overview of nonadiabatic dynamics simulations methods, with focus on the direct approach versus the fitting of potential energy surfaces. *Theor. Chem. Acc.* **133**, 1526 (2014).

[26] Thiel, W. Semiempirical quantum–chemical methods. *Wiley Interdiscip. Rev. Comput. Mol. Sci.* **4**, 145–157 (2014).

[27] Salvadori, G. *et al.* Protein control of photochemistry and transient intermediates in phytochromes. *Nat. Commun.* **13**, 6838 (2022).

[28] Toniolo, A., Ciminelli, C., Granucci, G., Laino, T. & Persico, M. QM/MM connection atoms for the multistate treatment of organic and biological molecules. *Theor. Chem. Acc.* **111**, 270–279 (2003).

[29] Stewart, J. J. Mopac2002 (2002). Fujitsu Limited: Tokyo, Japan.

[30] Rackers, J. A. *et al.* Tinker 8: Software tools for molecular design. *J. Chem. Theory Comput.* **14**, 5273–5289 (2018).

[31] Granucci, G. & Persico, M. Critical appraisal of the fewest switches algorithm for surface hopping. *J. Chem. Phys.* **126**, 134114 (2007).

[32] Hunter, J. D. Matplotlib: A 2d graphics environment. *Comput. Sci. Eng.* **9**, 90–95 (2007).

[33] Virtanen, P. *et al.* SciPy 1.0: Fundamental Algorithms for Scientific Computing in Python. *Nat. Methods* **17**, 261–272 (2020).

[34] Case, D. *et al.* Amber 2023 (2023). University of California, San Francisco.

[35] Case, D. A. *et al.* Ambertools. *J. Chem. Inf. Model* **63**, 6183–6191 (2023).

[36] Miao, Y., Feher, V. A. & McCammon, J. A. Gaussian Accelerated Molecular Dynamics: Unconstrained Enhanced Sampling and Free Energy Calculation. *J. Chem. Theory Comput.* **11**, 3584–3595 (2015).

[37] Frisch, M. J. *et al.* Gaussian 16 Revision A.03 (2016). Gaussian Inc. Wallingford CT.

[38] Bondanza, M., Nottoli, M., Cupellini, L., Lipparini, F. & Mennucci, B. Polarizable embedding QM/MM: the future gold standard for complex (bio)systems? *Phys. Chem. Chem. Phys.* **22**, 14433–14448 (2020).

[39] Shi, Y. *et al.* Polarizable atomic multipole-based amoeba force field for proteins. *J. Chem. Theory Comput* **9**, 4046–4063 (2013).

[40] Ren, P. & Ponder, J. W. Polarizable atomic multipole water model for molecular mechanics simulation. *J. Phys. Chem. B* **107**, 5933–5947 (2003).

[41] Ponder, J. W. *et al.* Current status of the amoeba polarizable force field. *J. Phys. Chem. B* **114**, 2549–2564 (2010).

[42] Liu, D. C. & Nocedal, J. On the limited memory bfgs method for large scale optimization. *Math. Program.* **45**, 503–528 (1989).

[43] Bussi, G., Donadio, D. & Parrinello, M. Canonical sampling through velocity rescaling. *J. Chem. Phys.* **126** (2007).

[44] Loco, D. *et al.* Hybrid QM/MM molecular dynamics with AMOEBA polarizable embedding. *J. Chem. Theory Comput* **13**, 4025–4033 (2017).

[45] Nottoli, M., Mennucci, B. & Lipparini, F. Excited state born–oppenheimer molecular dynamics through coupling between time dependent DFT and AMOEBA. *Phys. Chem. Chem. Phys.* **22**, 19532–19541 (2020).

[46] Nottoli, M. *et al.* QM/AMOEBA description of properties and dynamics of embedded molecules. *Wiley Interdiscip. Rev. Comput. Mol. Sci.* **13** (2023).

[47] Lagardère, L. *et al.* Tinker-hp: a massively parallel molecular dynamics package for multiscale simulations of large complex systems with advanced point dipole polarizable force fields. *Chem. Sci.* **9**, 956–972 (2018).

[48] Rackers, J. A. *et al.* Tinker 8: Software tools for molecular design. *J. Chem. Theory Comput.* **14**, 5273–5289 (2018).

[49] Frisch, M. J. *et al.* Gaussian development version, revision j.19 (2020). Gaussian, Inc., Wallingford CT, 2020.

[50] Invernizzi, M. & Parrinello, M. Rethinking metadynamics: From bias potentials to probability distributions. *J. Phys. Chem. Lett.* **11**, 2731–2736 (2020).

[51] Invernizzi, M. & Parrinello, M. Exploration vs convergence speed in adaptive-bias enhanced sampling. *J. Chem. Theory Comput.* **18**, 3988–3996 (2022).

[52] Nottoli, M., Bondanza, M., Lipparini, F. & Mennucci, B. An enhanced sampling QM/AMOEBA approach: The case of the excited state intramolecular proton transfer in solvated 3-hydroxyflavone. *J. Chem. Phys.* **154** (2021).

[53] The PLUMED consortium. Promoting transparency and reproducibility in enhanced molecular simulations. *Nat. Methods* **16**, 670–673 (2019).

[54] Tribello, G. A., Bonomi, M., Branduardi, D., Camilloni, C. & Bussi, G. Plumed 2: New feathers for an old bird. *Comput. Phys. Commun.* **185**, 604–613 (2014).

[55] Burgie, E. S., Zhang, J. & Vierstra, R. D. Crystal structure of deinococcus phytochrome in the photoactivated state reveals a cascade of structural rearrangements during photoconversion. *Struct.* **24**, 448–457 (2016).
