# Supplementary material for: Analogies and Differences in the Photoactivation Mechanism of Bathy and Canonical Bacteriophytochromes Revealed by Multiscale Modeling
Source: J Phys Chem Lett. 2024 Aug 1;15(31):8078–84. doi: 10.1021/acs.jpclett.4c01823 (PMC11376688; doi:10.1021/acs.jpclett.4c01823)
Supplement: Supplementary file 2 — jz4c01823_si_002.pdf [file jz4c01823_si_002.pdf]

jz-2024-01823x.R1

Name: Peer Review Information for "Analogies and Differences in the Photoactivation Mechanism of Bathy and Canonical Bacteriophytochromes Revealed by Multiscale Modeling"

First Round of Reviewer Comments

Reviewer: 1

Comments to the Author

The article by Salvadori and Mennucci reports an elegant and detailed multiscale computational investigation of the structural events that characterize the photoactivation mechanism of bathy and canonical bacteriophytochromes. The authors use state-of-the-art methods to explore the complexity of the multiple structural transitions that follow photoexcitation of the DB chromophore in these systems, including ground state and non-adiabatic QM/MM and MM dynamics including enhanced sampling methods.

The major advance reported in the paper is the evidence provided that both bathy and canonical bacteriophytochromes follow a similar photoisomerization hula-twist mechanism, in contrast to recent suggestions, despite the fact that the resting states are reversed in these systems. Overall, the work is nicely executed, reported and discussed, I have no technical concerns, and I recommend its publication in the Journal of Physical Chemistry Letters. Nevertheless, I indicate some minor points that the authors could address to clarify some aspects of their study:

1) It would be helpful to explain the experimental evidence that supports the fact that phytochromes pass through at least 2 intermediate states in the inactive-to-active transition.

2) In a related aspect, the key role of the protonation state of the C-ring propionic group deserves a more insightful explanation on why only in bathy phytochromes this is protonated, and how was this demonstrated. The authors indicate relevant references on this aspect, but a short justification would be appropiate in the article.

Reviewer: 2

Comments to the Author

The authors present a comprehensive computational investigation of the similarities between the interconversion of the Pr and Pfr states in canonical and bathy phytochromes. Through a multiscale approach spanning many timescales, the authors show that the photodynamics of both canonical and bathy phytochromes is initiated by a hula-twist motion in the bilin chromophore. The study is well written. I appreciate the authors' protocol design in this significant contribution to the field. I have only a

couple of minor questions for the authors, and I am fully committed to supporting the publication of their work after they are answered.

1) What is the rationale behind using Gaussian-accelerated MD? Is this because this technique is unconstrained and does not require any collective variables, or is it because a threshold on the height of the potential can be applied? OPES, in one of its variants, also allows for the setting of such a threshold.

2) In the SI, the authors show the result of using PCA to project the distances. PCA is known to identify the directions of the highest variance, not slow modes. Is there a reason the authors did not use, for instance, tICA?

3) Continuing my above comment, in Fig. S2, the clusters in the PCA space overlap. Could the authors also show the density (or free energy) to ensure that the clusters are properly separated?

Author's Response to Peer Review Comments:

Reviewer: 1

Comments:
The article by Salvadori and Mennucci reports an elegant and detailed multiscale computational investigation of the structural events that characterize the photoactivation mechanism of bathy and canonical bacteriophytochromes. The authors use state-of-the-art methods to explore the complexity of the multiple structural transitions that follow photoexcitation of the DB chromophore in these systems, including ground state and non-adiabatic QM/MM and MM dynamics including enhanced sampling methods.
The major advance reported in the paper is the evidence provided that both bathy and canonical bacteriophytochromes follow a similar photoisomerization hula-twist mechanism, in contrast to recent suggestions, despite the fact that the resting states are reversed in these systems. Overall, the work is nicely executed, reported and discussed, I have no technical concerns, and I recommend its publication in the Journal of Physical Chemistry Letters.

Authors' Response: We sincerely thank the reviewer for the very positive comment

Nevertheless, I indicate some minor points that the authors could address to clarify some aspects of their study:
1) It would be helpful to explain the experimental evidence that supports the fact that phytochromes pass through at least 2 intermediate states in the inactive-to-active transition.

Authors' Response: We thank the reviewer for the suggestion. Indeed, it is important to report that the intermediates are spectrally distinguishable and explain how vibrational spectroscopic techniques have played an important role in elucidating the mechanism of phytochrome activation.
In the introduction of the revised manuscript we have added the following paragraph:
"Thanks to vibrational spectroscopic techniques,[25] we know that along the inactive-to-active transition, phytochromes pass through at least two spectrally distinguishable intermediate states (Fig. 1),[26–30] namely Lumi(-R/F) and Meta(-R/F), although the exact number varies from phytochrome to phytochrome. This is because the D-ring-carbonyl stretching mode is very localized and environmentally sensitive, therefore it can be used as an excellent probe to follow changes in the interactions of the chromophore with the nearby residues."

2) In a related aspect, the key role of the protonation state of the C-ring propionic group deserves a more insightful explanation on why only in bathy phytochromes this is protonated, and how was this demonstrated. The authors indicate relevant references on this aspect, but a short justification would be appropiate in the article.

Authors' Response: In Nature Chem 7, 423–430 (2015), the authors performed difference (Pr minus Pfr and Meta-F minus Pfr) IR spectra for the fully protonated state (H2O), the partially deuterated state (H → D, dark), and the fully deuterated state (H → D, hv). They found a negative band at 1750 cm-1, then related to the Pfr state (insensitive to 13C isotope labeling), which underwent a downshift of 7 cm-1 upon H/D exchange in the dark, consistent with a stretching mode of the C=O group of a propionic acid chromophore side chain.

In the introduction of the revised manuscript we have added the following sentence:
"The latter also determines the protonation state of the C-ring propionic group (Cprop, Fig. 1), which infrared difference spectroscopy showed to be protonated in bathy phytochromes due to an unprecedentedly high pKa."

Reviewer: 2

Comments:
The authors present a comprehensive computational investigation of the similarities between the interconversion of the Pr and Pfr states in canonical and bathy phytochromes. Through a multiscale approach spanning many timescales, the authors show that the photodynamics of both canonical and bathy phytochromes is initiated by a hula-twist motion in the bilin chromophore. The study is well written. I appreciate the authors' protocol design in this significant contribution to the field. I have only a couple of minor questions for the authors, and I am fully committed to supporting the publication of their work after they are answered.

Authors' Response: We sincerely thank the reviewer for the very positive comment

1) What is the rationale behind using Gaussian-accelerated MD? Is this because this technique is unconstrained and does not require any collective variables, or is it because a threshold on the height of the potential can be applied? OPES, in one of its variants, also allows for the setting of such a threshold.

Authors' Response: We relied on the Gaussian accelerated MD technique because the main drawback of all CV-biasing approaches is the risk that the chosen CV space does not provide the most faithful representation of the true spectrum of metastable sub-ensembles and the barriers that separate them. Therefore, we wanted to use an unconstrained method to blindly explore the configuration space of the system without imposing a preferred direction.

2) In the SI, the authors show the result of using PCA to project the distances. PCA is known to identify the directions of the highest variance, not slow modes. Is there a reason the authors did not use, for instance, tICA?

Authors' Response: We did not use the tICA as our goal was to have structurally separated clusters to characterize different local environments around the chromophore.

3) Continuing my above comment, in Fig. S2, the clusters in the PCA space overlap. Could the authors also show the density (or free energy) to ensure that the clusters are properly separated?

Authors' Response: We apologize for the unclear presentation of the figure where labels of the panels were missing. We have added them in the revised figure together with densities for each of the clusters. These are structurally well separated as shown in Fig.2c where we report the KDE of selected distances. As it can be seen, in cluster 1, the D-ring carbonyl does not interact with a water molecule, but instead with Gln190. In both clusters 0 and 2, the D-ring carbonyl interacts with a water molecule, but in cluster 0 there is no hydrogen bond between the Cprop group and water molecules.
